# Recency-Weighted Statistical Modeling Approach to Attribute Illnesses Caused by 4 Pathogens to Food Sources Using Outbreak Data, United States

**DOI:** 10.3201/eid2701.203832

**Published:** 2021-01

**Authors:** Michael B. Batz, LaTonia C. Richardson, Michael C. Bazaco, Cary Chen Parker, Stuart J. Chirtel, Dana Cole, Neal J. Golden, Patricia M. Griffin, Weidong Gu, Susan K. Schmitt, Beverly J. Wolpert, Joanna S. Zablotsky Kufel, R. Michael Hoekstra

**Affiliations:** US Food and Drug Administration, College Park, Maryland, USA (M.B. Batz, M.C. Bazaco, C. Chen Parker, S.J. Chirtel, B.J. Wolpert);; Centers for Disease Control and Prevention, Atlanta, Georgia, USA (L.C. Richardson, D. Cole, P.M. Griffin, W. Gu, R.M. Hoekstra);; US Department of Agriculture, Washington, DC, USA (N.J. Golden, S.K. Schmitt, J.S. Zablotsky Kufel)

**Keywords:** foodborne diseases, disease outbreaks, food safety, Salmonella, Escherichia coli 0157, Listeria, Campylobacter, risk factors, models, statistical, analysis of variance

## Abstract

Foodborne illness source attribution is foundational to a risk-based food safety system. We describe a method for attributing US foodborne illnesses caused by nontyphoidal *Salmonella enterica*, *Escherichia coli* O157, *Listeria monocytogenes,* and *Campylobacter* to 17 food categories using statistical modeling of outbreak data. This method adjusts for epidemiologic factors associated with outbreak size, down-weights older outbreaks, and estimates credibility intervals. On the basis of 952 reported outbreaks and 32,802 illnesses during 1998–2012, we attribute 77% of foodborne *Salmonella* illnesses to 7 food categories (seeded vegetables, eggs, chicken, other produce, pork, beef, and fruits), 82% of *E. coli* O157 illnesses to beef and vegetable row crops, 81% of *L. monocytogenes* illnesses to fruits and dairy, and 74% of *Campylobacter* illnesses to dairy and chicken. However, because *Campylobacter* outbreaks probably overrepresent dairy as a source of nonoutbreak campylobacteriosis, we caution against using these *Campylobacter* attribution estimates without further adjustment.

Each year in the United States, nontyphoidal *Salmonella*, *Escherichia coli* O157, *Listeria monocytogenes,* and *Campylobacter* cause >2 million estimated foodborne illnesses, 31,000 hospitalizations, and 700 deaths (*1*), representing an estimated $9–$11 billion in impacts to human health (*2*,*3*). Estimating the percentage of these illnesses attributable to the consumption of specific foods (i.e., foodborne illness source attribution) is foundational to a risk-based national food safety system (*4*). Such estimates can inform strategic planning, priority setting, risk assessments, economic analyses, and evaluations of the impacts of regulations and interventions (*5*).

Numerous studies in the United States and worldwide have estimated source attribution on the basis of aggregated foodborne outbreak data (*6*–*12*). For the United States, the Centers for Disease Control and Prevention (CDC) previously estimated the number of domestically acquired foodborne illnesses, hospitalizations, and deaths attributable to food categories based on analysis of outbreaks during 1998–2008 (*13*).

Through the Interagency Food Safety Analytics Collaboration (IFSAC), CDC, the US Food and Drug Administration, and the US Department of Agriculture Food Safety and Inspection Service work in partnership to develop improved source attribution estimates through multiple interconnected projects (*14*,*15*). This study reflects a tri-agency effort to update and harmonize estimates for the United States for nontyphoidal *Salmonella*, *E. coli* O157, *L. monocytogenes,* and *Campylobacter* using data from outbreaks that occurred during 1998–2012.

IFSAC’s approach addresses some of the limitations of prior studies. We describe this method here. We use statistical modeling to mitigate the influence of large outbreaks that might bias estimates, and we incorporate epidemiologic factors relevant to outbreak size. We weight recent outbreaks more heavily than older ones and quantify uncertainty by estimating credibility intervals around estimates. We also use an updated food categorization scheme that better meets the needs of the regulatory agencies.

## Methods

### Data Sources

CDC’s Foodborne Disease Outbreak Surveillance System (FDOSS) collects standardized reports submitted by state, local, and territorial health departments on foodborne disease outbreaks. In FDOSS, outbreaks are defined as the occurrence of >2 cases of a similar illness resulting from the ingestion of a common food (*16*). We extracted data from FDOSS on reported foodborne outbreaks caused by nontyphoidal *Salmonella enterica*, *E. coli* O157 (*E. coli* O157:H7 and *E. coli* O157:NM), *L. monocytogenes,* and *Campylobacter* spp., in which the first illness occurred in a US state or the District of Columbia during 1998–2012. We extracted data on December 18, 2013. Analysis was conducted by using SAS 9.3, JMP Pro (SAS Institute, https://www.sas.com), and R (R Foundation for Statistical Computing, https://www.r-project.org).

We included only outbreaks with a single causal pathogen and for which implicated foods could be assigned to a single food category because those outbreaks have the clearest information. We excluded outbreaks caused by multiple pathogens or for which no food or ingredient was implicated, including outbreaks with a complex food vehicle (i.e., consisting of ingredients belonging to >1 food category) for which the implicated ingredient was not determined. A previously published method (*13*) for assigning the food category for complex food outbreaks could not be applied to more recent data without substantial revision.

We excluded outbreaks for which implicated foods came from >1 food category (i.e., multiple foods). For example, an outbreak for which apples and cantaloupe were both implicated would be included because both fall into the fruits category, but an outbreak for which apples and cheese were both implicated would be excluded.

By using a hierarchical scheme of 22 food categories, we assigned outbreaks to a single category on the basis of implicated foods or ingredients (*17*). Because of sparse data, outbreaks in 8 food categories were aggregated into 3 combined categories: other meat and poultry (other meat, other poultry); other seafood (shellfish, other aquatic animals); and other produce (fungi, herbs, root-underground, nuts-seeds), resulting in 17 food categories for our analysis (Appendix Figure 1).

In FDOSS, an outbreak must have >2 ill persons (*16*). For *Salmonella*, *E. coli* O157, and *Campylobacter*, outbreaks with confirmed etiology are defined as those in which the outbreak strain was isolated from >2 patients or from epidemiologically implicated food; confirmed outbreaks of *L. monocytogenes* infections must have 1 person with the outbreak strain isolated from a normally sterile site (*18*). (Cases of listeriosis can also be diagnosed based on symptoms and culture of pregnancy-associated products of conception, which are not sterile.) The etiology of an outbreak not meeting these conditions is considered to be suspected. We found no statistically significant differences in outbreak size or foods implicated between outbreaks with confirmed and those with suspected status, and therefore included outbreaks with suspected etiology in the analysis (Appendix). The final dataset used for exploratory analysis and estimates of sources included 952 outbreaks assigned to 17 food categories (Table 1).

**Table 1:** Number of outbreaks and outbreak illnesses caused by a single pathogen and due to a single food category for *Salmonella, Escherichia coli* O157, *Listeria monocytogenes,* and *Campylobacter*, Foodborne Disease Outbreak Surveillance System, United States, 1998–2012*

Food category	Nontyphoidal *Salmonella* spp.	*E. coli* O157	*Listeria monocytegenes*	*Campylobacter* spp.	Total
Beef	47 (1,473)	97 (1,813)	1 (4)	2 (5)	147 (3,295)
Pork	51 (1,098)	0	2 (11)	1 (27)	54 (1,136)
Chicken	114 (2,648)	1 (36)	1 (3)	24 (230)	140 (2,917)
Turkey	49 (1,308)	1 (2)	4 (124)	5 (44)	59 (1,478)
Other meat or poultry	6 (84)	2 (9)	0	2 (6)	10 (99)
Game	2 (8)	4 (18)	0	1 (2)	7 (28)
Dairy	24 (793)	18 (399)	12 (124)	106 (3,395)	160 (4,711)
Eggs	140 (5,245)	0	0	0	140 (5,245)
Fish	12 (286)	0	0	1 (3)	13 (289)
Other seafood	4 (36)	0	0	5 (344)	9 (380)
Grains, beans	7 (268)	0	0	0	7 (268)
Oils, sugars	0	0	0	1 (3)	1 (3)
Fruits	46 (2,510)	11 (893)	1 (147)	2 (29)	60 (3,579)
Seeded vegetables	34 (4,001)	0	0	3 (136)	37 (4,137)
Sprouts	33 (1,266)	6 (55)	2 (26)	0	41 (1,347)
Vegetable row crops	10 (412)	29 (1,029)	1 (10)	7 (372)	47 (1,823)
Other produce	18 (1,923)	1 (8)	0	1 (136)	20 (2,067)
Total	597 (23,359)	170 (4,262)	24 (449)	161 (4,732)	952 (32,802)

*Number of outbreak-associated illnesses in parentheses. Nontyphoidal *Salmonella* is divided into *S. enterica* serovar Enteritidis and other serovars (Appendix Table 2).

### Exploratory Analysis

We focused exploratory analysis on factors influencing outbreak size. We used the total number of reported illnesses as the measure of outbreak size. Whereas most outbreaks are small, some are very large. For example, of the 4,732 reported *Campylobacter* outbreak illnesses during the entire 15-year period, more than one third (1,644) were from a single outbreak. Large outbreaks might not be representative of the sources of sporadic illnesses and might overly influence estimates of food sources (*13*).

**Figure 1:**
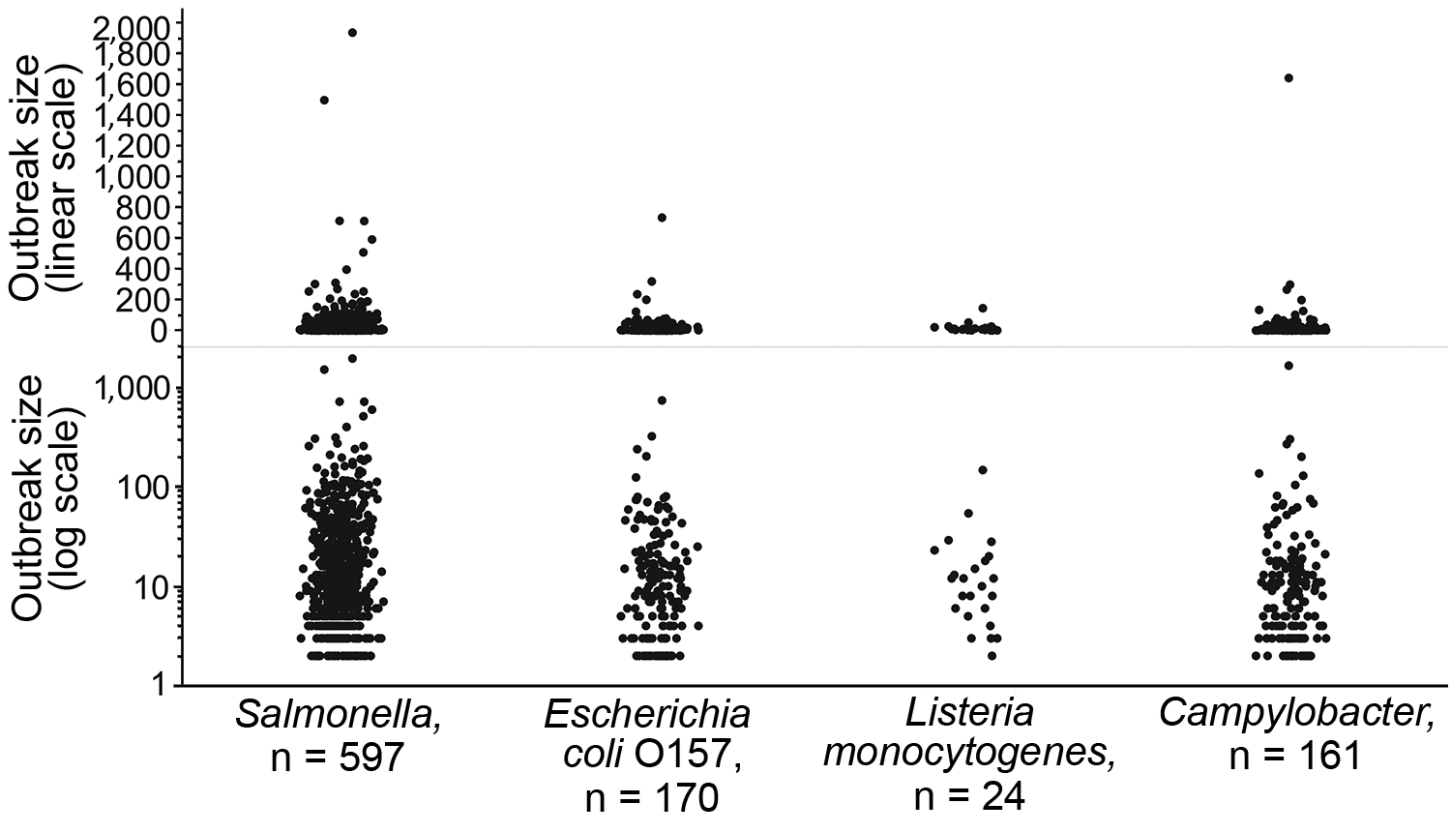
Number of reported illnesses for foodborne disease outbreaks caused by a single pathogen and attributable to a single food category, using linear and log scales, for *Salmonella*, *Escherichia coli* O157, *Listeria monocytogenes*, and *Campylobacter*, Foodborne Disease Outbreak Surveillance System, United States, 1998–2012.

Untransformed outbreak size is skewed and varies across pathogens (Figure 1). Log transformation of outbreak size results in distributions that are more symmetric and normally distributed, although considerable variation remains within and across pathogens (Figure 1). We therefore used log-transformed outbreak size in statistical modeling.

FDOSS data include epidemiologic factors that might relate to the size and scope of an outbreak, including pathogen, number of states in which outbreak exposures occurred, implicated foods and ingredients, and the type of location in which food was prepared (e.g., restaurant or private home). We explored the relationships between outbreak size and these variables.

**Figure 2:**
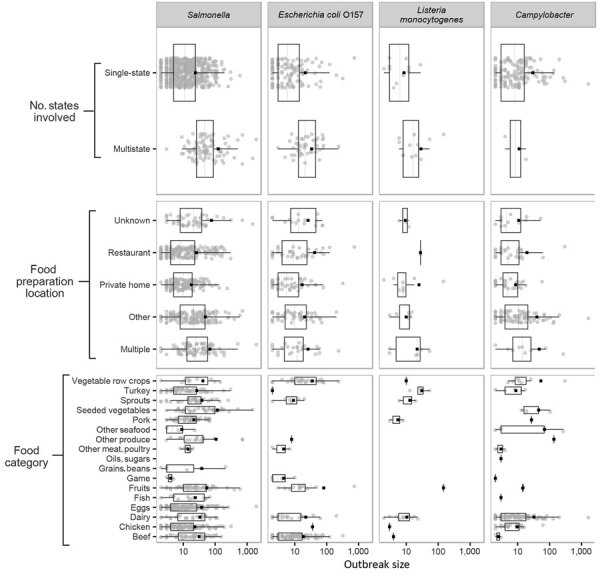
Number of reported illnesses (log scale) for foodborne disease outbreaks caused by a single pathogen and attributable to a single food category, for 3 outbreak characteristics, for *Salmonella *(A)*, Escherichia coli *O157 (B)*, Listeria monocytogenes *(C)*, and Campylobacter *(D), Foodborne Disease Outbreak Surveillance System, United States, 1998–2012. Each panel displays outbreak size for a given pathogen, grouped by 1 of 3 categorical variables. Each includes a scatterplot of individual outbreaks (indicated by solid circles), the mean (indicated by solid squares), and a boxplot showing median, interquartile range, and minimum and maximum values inside the inner and outer fences (1.5 interquartile range).

Distinct differences in distributions of outbreak size can be observed by pathogen and 3 categorical variables: food category, type of food preparation location, and whether exposures occurred in multiple states or a single state (Figure 2). For example, the mean size of multistate outbreaks is larger than single-state outbreaks for *Salmonella*, *E. coli* O157, and *L. monocytogenes*. Differences in grouped means can be observed for all 3 categorical variables for *L. monocytogenes* despite the small number of outbreaks.

### Statistical Modeling

Whereas prior studies calculated attribution proportions on the basis of observed counts of reported outbreak events or outbreak illnesses (*6*,*13*), in this study we developed a model-based approach to estimate the number of outbreak illnesses for attribution. This approach mitigates the impact of large outbreaks and enables the incorporation of epidemiologic factors beyond pathogen and food category.

After considering several approaches, we chose analysis of variance (ANOVA) of log-transformed outbreak sizes as the modeling framework, partly based on simplicity and interpretability (Appendix). For each pathogen, we developed a model to estimate the log-transformed number of illnesses based on the 3 factors shown to be associated with outbreak size: food category, type of food preparation location, and whether exposures occurred in multiple states or a single state. Each of these factors was found through 1-way ANOVA to be a statistically significant (p<0.05) predictor of outbreak size for >1 pathogens. Although not all 3 factors were significant for all pathogens, we included them to maintain uniformity across the analysis. We explored serotype-specific ANOVA models for *Salmonella*, but for most serotypes these models did not find different distributions of outbreaks across food categories or meaningful differences in outbreak size across the other factors. The exception was serotype Enteritidis, outbreaks of which did display differences from other serotypes. Therefore, we developed 2 distinct *Salmonella* ANOVA models: 1 for Enteritidis and 1 for all other serotypes.

Each of the 5 pathogen-specific models estimates the log-transformed number of illnesses for each reported outbreak on the basis of that outbreak’s characteristics as defined by the categorical variables. We then back-transformed the model-estimated numbers of illnesses (*e* raised to the transformed values) and summed the 2 sets of *Salmonella* estimates. Additional information on model selection and fit is presented in the Appendix.

As expected, ANOVA models reduce variation in outbreak size and the influence of very large outbreaks. This effect is shown in Appendix Figure 3, which compares the number of reported illnesses with the number of model-estimated illnesses. The figure also shows the wide variation in the number of outbreaks for different pathogen–food category pairs.

### Recency Weighting

Because of changes over time in food consumption patterns, food production and processing practices, food safety activities, regulatory interventions, and other factors, recent outbreaks are probably more representative of current foodborne illness attribution than older outbreaks. We explored estimating attribution on the basis of only 3, 5, or 7 years of the most recent outbreaks, but data sparseness and high year-to-year variability, particularly in food categories for which outbreaks were not reported every year, led to instability and more statistical uncertainty in attribution estimates when older outbreaks were excluded (Appendix). Therefore, we included older outbreaks but down-weighted them on the basis of recency. Outbreaks older than 5 years were multiplied by an exponential decay function, an approach long used in many fields to down-weight older data in forecasting and time-series models, including public health surveillance (*19*,*20*). This approach is flexible to inclusion of additional years of data; as the number of years of data increases, the earliest years have less and less weight.

**Figure 4:**
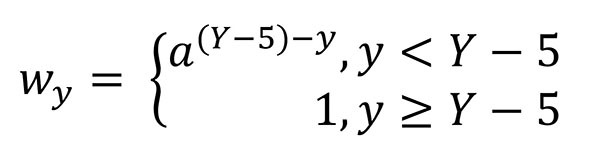
Equation. The multiplicative recency-weighting factor *w* for an outbreak in year *y* is defined as a function of decay parameter *a* and the most recent year of data *Y.*

The multiplicative recency-weighting factor *w* for an outbreak in year *y* is defined as a function of decay parameter *a* and the most recent year of data *Y* (Equation, Figure 4). We used a decay parameter *a* of 5/7 (0.7142). This factor resulted in outbreaks occurring during 2008–2012 providing 67% of the overall information, with 28% from outbreaks occurring during 2003–2007, and 5% outbreaks occurring during 1998–2002 (Appendix Table 4).

### Calculating Attribution Percentages

**Figure 5:**
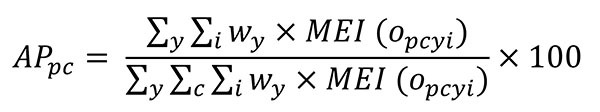
Equation. The estimated attribution percentage.

For pathogen *p* and food category *c*, the attribution percentage *AP_pc_* is calculated by dividing the sum of recency-weighted model-estimated illnesses of that pathogen–food category pair across all years by the sum of recency-weighted model-estimated illnesses for all food categories associated with that pathogen for all years. The estimated attribution percentage *AP_pc_* is defined as (Equation, Figure 5) where *MEI* (*o_pcyi_*) is the number of model-estimated illnesses for a specific outbreak *o_i_* and *i* is the instance in the set of outbreaks associated with a pathogen–food pair occurring in a given year. To estimate 90% credibility intervals for each *AP_pc_*, Bayesian bootstrap resampling (10,000 per pathogen–food category pair) was performed on the weighted model estimates (*21*,*22*). The 5th and 95th percentiles of the bootstrap distributions were used as the lower and upper bounds for the credibility intervals.

We conducted sensitivity analyses to examine the impacts of data selection and modeling choices on estimates. These analyses included comparing our attribution estimates to those based on reported outbreak illnesses and log-transformed illnesses, evaluating the impact of alternate ANOVA model specifications, examining the impact of recency-weighting choices and approaches, and evaluating the impact of particularly large and influential outbreaks.

## Results

We extracted data on 2,732 US outbreaks caused by nontyphoidal *Salmonella*, *E. coli* O157, *L. monocytogenes*, and *Campylobacter* that occurred during 1998–2012. We excluded 77 outbreaks because they were caused by multiple pathogens, excluded an additional 1,014 because they did not have an identified food vehicle, and excluded an additional 689 that could not be assigned to a single food category (Appendix Figure 2). These exclusions resulted in a dataset with 952 outbreaks (35% of 2,732), each caused by a single pathogen and assignable to 1 of 17 food categories (Table 1).

**Figure 3:**
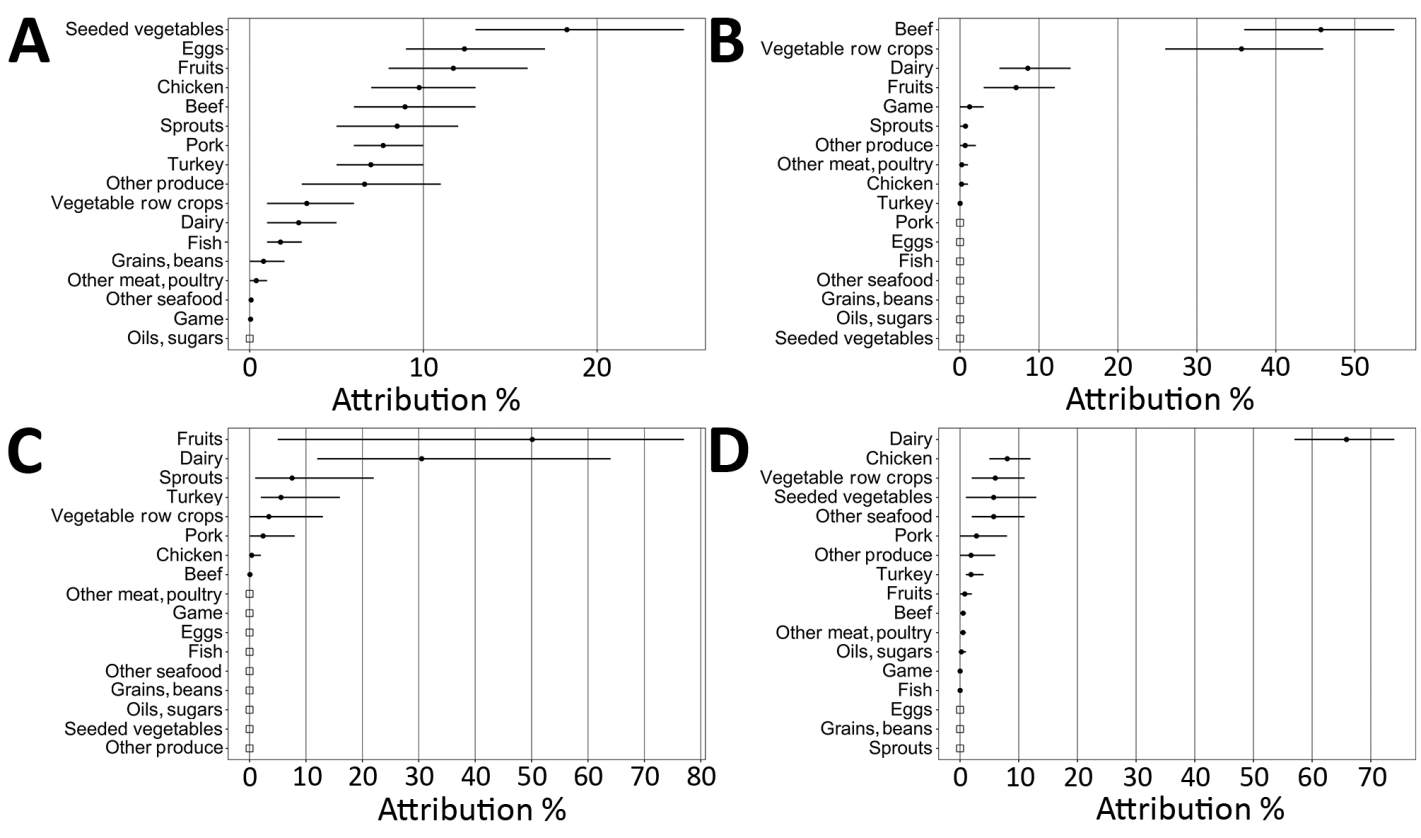
Estimated percentages of foodborne illnesses attributed to food categories and 90% credibility intervals (error bars) for *Salmonella, Escherichia coli *O157*, Listeria monocytogenes, and Campylobacter*, based on analysis of single-pathogen, single-food category outbreaks, Foodborne Disease Outbreak Surveillance System, United States, 1998–2012. Percentages are presented in descending order. Open squares indicate that no illnesses were attributed to that food category because no outbreaks were reported for that pathogen in that food category during the study period. Estimates calculated by using analysis of variance model–estimated outbreak illnesses for single pathogen, single food category outbreaks occurring during 1998–2012, with down-weighting of outbreaks that occurred during 1998–2007.

The final estimates (Table 2; Figure 3) attributed *Salmonella* illnesses more broadly than other pathogens, with nonzero estimates for 16 categories; of those, 4 food categories had estimated percentages >10%: seeded vegetables (e.g., tomatoes), eggs, fruits, and chicken. Cumulatively, the top 7 food categories accounted for 77% of illnesses. Credibility intervals for *Salmonella* were largely overlapping but comparatively narrow, attributable in part to the high number of *Salmonella* outbreaks in the analysis. In contrast, 82% of illnesses caused by *E. coli* O157 were attributed to only 2 food categories, beef and vegetable row crops (e.g., leafy greens). Only 2 other food categories, dairy and fruits, had estimated attribution percentages >1%. Similarly, 81% of illnesses caused by *L. monocytogenes* were attributed to 2 food categories, dairy and fruits. Only 4 other food categories (sprouts, turkey, vegetable row crops, and pork) had estimated attribution percentages >1%. The wide and overlapping credibility intervals reflect the very small number of *L. monocytogenes* outbreaks in the analysis (n = 24).

**Table 2:** Estimated percentages of foodborne illnesses attributed to 17 food categories and 90% credibility intervals for *Salmonella*, *Escherichia coli* O157, *Listeria monocytogenes*, and *Campylobacter*, based on analysis of single pathogen, single food category outbreaks, Foodborne Disease Outbreak Surveillance System, 1998–2012*

Food category	% (90% credibility interval)			
	*Salmonella*	*E. coli* O157	*L. monocytogenes*	*Campylobacter*
Land animals				
Beef	9 (6–13)	46 (36–55)	0 (0–1)	1 (<1–1)
Pork	8 (6–10)	–	2 (<1–8)	3 (<1–8)
Chicken	10 (7–13)	0 (0–1)	0 (0–2)	8 (5–12)
Turkey	7 (5–10)	0 (0–<1)	6 (2–16)	2 (1–4)
Other meat or poultry	0 (<1–1)	0 (0–1)	–	1 (<1–1)
Game	0 (0–<1)	1 (<1–3)	–	0 (0–<1)
Dairy	3 (1–5)	9 (5–14)	31 (12–64)	66 (57–74)
Eggs	12 (9–17)	–	–	–
Aquatic animals				
Fish	2 (1–3)	–	–	0 (0–<1)
Other seafood	0 (0–<1)	–	–	6 (2–11)
Plants				
Grains, beans	1 (<1–2)	–	–	–
Oils, sugars	–	–	–	0 (0–1)
Fruits	12 (8–16)	7 (3–12)	50 (5–77)	1 (<1–2)
Seeded vegetables	18 (13–25)	–	–	6 (1–13)
Sprouts	8 (5–12)	1 (<1–1)	8 (1–22)	–
Vegetable row crops	3 (1–6)	36 (26–46)	3 (<1–13)	6 (2–11)
Other produce	7 (3–11)	1 (0–2)	–	2 (<1–6)

*Estimates calculated by using analysis of variance model–estimated outbreak illnesses for single pathogen, single food category outbreaks occurring during 1998–2012, with down-weighting of outbreaks that occurred during 1998–2007. Because of rounding, 0 indicates nonzero estimates <0.5. Dashes indicate pathogen–food category pairs for which we did not estimate attribution percentages because of a lack of outbreaks.

An estimated 66% of *Campylobacter* outbreak illnesses were attributed to the dairy category. This percentage was substantially higher than for any other food category. About 8% of illnesses are attributed to chicken; 6% were attributed respectively to vegetable row crops, seeded vegetables, and other seafood.

We found estimates to be robust across a wide variety of scenarios. We assessed the sensitivity of estimates to particularly influential outbreaks and to modeling decisions, such as choices in statistical modeling, down-weighting of older outbreaks, and consideration of etiology status (Appendix).

## Discussion

Although only a small proportion of foodborne illnesses are part of recognized outbreaks, outbreak investigations can provide insights into the causes and contributing factors leading to infection. Because linking an illness to a particular food is rarely possible except during an outbreak, aggregated data from foodborne outbreaks have been used to estimate the food sources of all illnesses caused by specific pathogens in numerous countries (*6*–*12*).

Whereas our approach addresses numerous challenges with estimating attribution percentages on the basis of outbreak data, some issues must be considered when using these estimates to inform food safety decision-making. Our analysis does not indicate the point of contamination, because outbreak investigations implicate only the food vehicle that was consumed. Moreover, the outbreaks included in this analysis include only 35% of the reported foodborne disease outbreaks caused by these pathogens during the study period, and they might not be representative of all foodborne outbreaks caused by these pathogens. The exclusion of outbreaks attributable to complex foods for which the contaminated ingredient was not determined could result in underrepresentation of food categories containing foods often eaten as part of complex dishes (e.g., leafy greens and eggs) (*23*). However, because the published method for assigning food categories to these complex food outbreaks is somewhat subjective and relies on internet searches for recipes (*24*), excluding these outbreaks provides results based on the most accurate available data. A method to incorporate data from these outbreaks is being developed.

Foods are implicated in outbreaks through epidemiologic analyses, by isolation of the causal pathogen from implicated food, through examination of supply chain records or environmental assessments, or by other information. The strength of evidence implicating foods varies widely across outbreaks.

For some pathogens and pathogen–food pairs, the number of outbreaks available for analyses was quite low. For example, our data include only 24 outbreaks caused by *L. monocytogenes*, so 1 fruits-linked outbreak (a very large outbreak that occurred in 2011 and was associated with contaminated cantaloupe) had a profound influence on attribution estimates for this pathogen. However, our approach reflects the uncertainties associated with sparse data in wider credibility intervals.

Although we weighted recent outbreaks more heavily than older ones because recent outbreaks are probably more representative of current attribution, we did not formally account for possible changes in underlying factors over time in the main effects model. Examples of such factors could include changes in pathogen-specific disease incidence, outbreak investigation practices, or outbreak reporting by states. Generalizing outbreak-based attribution to overall foodborne disease assumes, implicitly, that the foods implicated in outbreaks reflect the food sources of illness in the general population. However, these assumptions might not always hold. For example, ≈10% of outbreaks occurred among institutionalized populations, such as those in correctional facilities, hospitals, and nursing homes. In these populations, case-ascertainment rates, food options, and sources of food contamination might not be representative of the general population. However, such outbreaks might elucidate setting- or subpopulation-specific contamination problems that are difficult to identify among the general population.

*Campylobacter* attribution presents a specific challenge. Our outbreak-based model attributes 66% (90% credibility interval 57%–74%) of foodborne campylobacteriosis to dairy, which is in line with other outbreak-based estimates for the United States (*6*,*13*). However, most foodborne *Campylobacter* outbreaks in this study were associated with unpasteurized fluid milk, which is not widely consumed by the general population. For example, in a Foodborne Active Surveillance Network population survey of food exposures, only 3% reported consuming unpasteurized milk in the preceding week (*25*). Moreover, outbreak-based estimates are not consistent with other lines of evidence. An analysis of 38 case–control studies of sporadic campylobacteriosis found a much smaller percentage of illnesses attributable to consumption of raw milk than chicken (*12*). For example, 1 of these studies, a Foodborne Active Surveillance Network case–control study, attributed 1.5% of campylobacteriosis cases to consumption of unpasteurized milk, compared with 24% to consumption of chicken prepared in a restaurant (*26*). Structured expert judgment studies conducted in the United States and in other countries estimate 8%–10% of foodborne campylobacteriosis to be attributable to dairy products (principally, raw milk), compared with 33%–72% to chicken (*27*–*30*).

Because *Campylobacter* outbreaks appear to overrepresent dairy as a source of sporadic *Campylobacter* illness, we do not advise using these attribution percentages without further adjustment or without considering additional information. Removing the dairy category entirely might be an appropriate adjustment, given that the resulting distribution of *Campylobacter* attribution estimates across other food categories is more consistent with the published literature (*31*). When the dairy category is excluded from this analysis, 29% of *Campylobacter* illnesses are attributed to poultry (23.5% to chicken and 5.5% to turkey), 18% to vegetable row crops, 17% to seeded vegetables, 17% to other seafood, 8% to pork, 6% to other produce, and 6% to other food categories.

Our estimates reflect data on outbreaks that occurred during 1998–2012 because those were the most recent data available at the outset of this effort. We do not include more recent outbreaks in this analysis because substantial preparation of the data was needed, and because the primary purpose of this report is to describe our methods and explain modeling decisions. IFSAC has published reports based on more recent outbreaks using the methodology described in this article (*32*).

To address some of the challenges with using outbreak data to estimate the food categories responsible for foodborne illnesses, we developed an approach that reduces the influence of large outbreaks, adjusts for important epidemiologic characteristics, and weights recent data more heavily than older data. We also incorporate an updated food categorization scheme better aligned to the needs of regulatory agencies and provide statistical uncertainty around the estimates. This approach can be used for routine updating of estimates by incorporating additional years of data.

The resulting estimates of attribution percentages for *Salmonella*, *E. coli* O157, *L. monocytogenes*, and *Campylobacter* can play an important role in science- and risk-based decision-making because they can be used alongside other data to inform regulatory decisions, to prioritize food safety efforts, and to evaluate the effectiveness of prevention measures. Further, federal agency consensus on a single set of outbreak-based attribution estimates improves the transparency of governmental efforts to inform and engage stakeholders, such as industry and consumers, about food safety strategies.

Appendix Additional information about recency-weighted statistical modeling approach to attribute illnesses caused by 4 pathogens to food sources using outbreak data, United States.

## References

[1] Scallan E, Hoekstra RM, Angulo FJ, Tauxe RV, Widdowson MA, Roy SL, et al. Foodborne illness acquired in the United States—major pathogens. Emerg Infect Dis. 2011;17:7–15. 10.3201/eid1701.P11101 21192848 PMC3375761

[2] Hoffmann S, Maculloch B, Batz MB. Economic burden of major foodborne illnesses acquired in the United States. Washington: US Department of Agriculture Economic Research Service; 2015. Economic Information Bulletin No. (EIB-140) [cited 2018 Jun 6]. https://www.ers.usda.gov/publications/pub-details/?pubid=43987

[3] Minor T, Lasher A, Klontz K, Brown B, Nardinelli C, Zorn D. The per case and total annual costs of foodborne illness in the United States. Risk Anal. 2015;35:1125–39. 10.1111/risa.12316 25557397

[4] Institute of Medicine and National Research Council Committee on the Review of Food and Drug Administration’s Role in Ensuring Safe Food. Enhancing food safety: the role of the Food and Drug Administration. Wallace RB, Oria M, editors. Washington: National Academies Press; 2010. 25032388

[5] Mangen MJ, Batz MB, Käsbohrer A, Hald T, Morris JG Jr, Taylor M, et al. Integrated approaches for the public health prioritization of foodborne and zoonotic pathogens. Risk Anal. 2010;30:782–97. 10.1111/j.1539-6924.2009.01291.x 19765248

[6] Batz MB, Hoffmann S, Morris JG Jr. Ranking the disease burden of 14 pathogens in food sources in the United States using attribution data from outbreak investigations and expert elicitation. J Food Prot. 2012;75:1278–91. 10.4315/0362-028X.JFP-11-418 22980012

[7] Greig JD, Ravel A. Analysis of foodborne outbreak data reported internationally for source attribution. Int J Food Microbiol. 2009;130:77–87. 10.1016/j.ijfoodmicro.2008.12.031 19178974

[8] Jackson BR, Griffin PM, Cole D, Walsh KA, Chai SJ. Outbreak-associated *Salmonella enterica* serotypes and food Commodities, United States, 1998-2008. Emerg Infect Dis. 2013;19:1239–44. 10.3201/eid1908.121511 23876503 PMC3739514

[9] King N, Lake R, Campbell D. Source attribution of nontyphoid salmonellosis in new zealand using outbreak surveillance data. J Food Prot. 2011;74:438–45. 10.4315/0362-028X.JFP-10-323 21375881

[10] Pires SM, Vigre H, Makela P, Hald T. Using outbreak data for source attribution of human salmonellosis and campylobacteriosis in Europe. Foodborne Pathog Dis. 2010;7:1351–61. 10.1089/fpd.2010.0564 20586609

[11] Ravel A, Greig J, Tinga C, Todd E, Campbell G, Cassidy M, et al. Exploring historical Canadian foodborne outbreak data sets for human illness attribution. J Food Prot. 2009;72:1963–76. 10.4315/0362-028X-72.9.1963 19777901

[12] Domingues AR, Pires SM, Halasa T, Hald T. Source attribution of human campylobacteriosis using a meta-analysis of case-control studies of sporadic infections. Epidemiol Infect. 2012;140:970–81. 10.1017/S0950268811002676 22214729

[13] Painter JA, Hoekstra RM, Ayers T, Tauxe RV, Braden CR, Angulo FJ, et al. Attribution of foodborne illnesses, hospitalizations, and deaths to food commodities by using outbreak data, United States, 1998-2008. Emerg Infect Dis. 2013;19:407–15. 10.3201/eid1903.111866 23622497 PMC3647642

[14] Interagency Food Safety Analytics Collaboration. About IFSAC. 2017 [cited 2017 Aug 7]. https://www.cdc.gov/foodsafety/ifsac/overview/index.html

[15] Ebel ED, Williams MS, Cole D, Travis CC, Klontz KC, Golden NJ, et al. Comparing characteristics of sporadic and outbreak-associated foodborne illnesses, United States, 2004–2011. Emerg Infect Dis. 2016;22:1193–200. 10.3201/eid2207.150833 27314510 PMC4918141

[16] Gould LH, Walsh KA, Vieira AR, Herman K, Williams IT, Hall AJ, et al.; Centers for Disease Control and Prevention. Surveillance for foodborne disease outbreaks - United States, 1998-2008. MMWR Surveill Summ. 2013;62:1–34. 23804024

[17] Richardson LC, Bazaco MC, Parker CC, Dewey-Mattia D, Golden N, Jones K, et al. An updated scheme for categorizing foods implicated in foodborne disease outbreaks: a tri-agency collaboration. Foodborne Pathog Dis. 2017;14:701–10. 10.1089/fpd.2017.2324 28926300 PMC6317073

[18] Centers for Disease Control and Prevention. Guide to confirming an etiology in foodborne disease outbreak. 2015 [cited 2018 Jul 12]. https://www.cdc.gov/foodsafety/outbreaks/investigating-outbreaks/confirming_diagnosis.html

[19] Brown RG. Smoothing, forecasting and prediction of discrete time series. Englewood Cliffs (NJ): Prentice-Hall; 1963.

[20] Ngo L, Tager IB, Hadley D. Application of exponential smoothing for nosocomial infection surveillance. Am J Epidemiol. 1996;143:637–47. 10.1093/oxfordjournals.aje.a008794 8610681

[21] Rubin DB. The Bayesian bootstrap. Ann Stat. 1981;9:130–4. 10.1214/aos/1176345338

[22] Davison AC, Hinkley DV. Bootstrap methods and their application. Cambridge and New York: Cambridge University Press; 1997.

[23] St Louis ME, Morse DL, Potter ME, DeMelfi TM, Guzewich JJ, Tauxe RV, et al. The emergence of grade A eggs as a major source of *Salmonella enteritidis* infections. New implications for the control of salmonellosis. JAMA. 1988;259:2103–7. 10.1001/jama.1988.03720140023028 3279240

[24] Painter JA, Ayers T, Woodruff R, Blanton E, Perez N, Hoekstra RM, et al. Recipes for foodborne outbreaks: a scheme for categorizing and grouping implicated foods. Foodborne Pathog Dis. 2009;6:1259–64. 10.1089/fpd.2009.0350 19968563

[25] Centers for Disease Control and Prevention. Foodborne Active Surveillance Network (FoodNet) population survey atlas of exposure, 2006–2007. Atlanta: US Department of Health and Human Services, Centers for Disease Control and Prevention; 2011 [cited 2019 Dec 12]. https://www.cdc.gov/foodnet/surveys/foodnetexposureatlas0607_508.pdf

[26] Friedman CR, Hoekstra RM, Samuel M, Marcus R, Bender J, Shiferaw B, et al.; Emerging Infections Program FoodNet Working Group. Risk factors for sporadic Campylobacter infection in the United States: A case-control study in FoodNet sites. Clin Infect Dis. 2004;38(Suppl 3):S285–96. 10.1086/381598 15095201

[27] Hoffmann S, Fischbeck P, Krupnick A, McWilliams M. Using expert elicitation to link foodborne illnesses in the United States to foods. J Food Prot. 2007;70:1220–9. 10.4315/0362-028X-70.5.1220 17536683

[28] Havelaar AH, Galindo AV, Kurowicka D, Cooke RM. Attribution of foodborne pathogens using structured expert elicitation. Foodborne Pathog Dis. 2008;5:649–59. 10.1089/fpd.2008.0115 18687052

[29] Tam CC, Larose T, O’Brien SJ. Costed extension to the Second Study of Infectious Intestinal Disease in the Community: identifying the proportion of foodborne disease in the UK and attributing foodborne disease by food commodity. Liverpool (UK): University of Liverpool; 2014. Project B18021 (FS231043) [cited 2019 Dec 12]. https://livrepository.liverpool.ac.uk/3014609

[30] Butler AJ, Pintar KD, Thomas MK. Estimating the relative role of various subcategories of food, water, and animal contact transmission of 28 enteric diseases in Canada. Foodborne Pathog Dis. 2016;13:57–64. 10.1089/fpd.2015.1957 26863428 PMC4796518

[31] Interagency Food Safety Analytics Collaboration. Foodborne illness source attribution estimates for 2013 for *Salmonella, Escherichia coli* O157, *Listeria monocytogenes*, and *Campylobacter* using multi-year outbreak surveillance data, United States. Atlanta and Washington: Centers for Disease Control and Prevention, US Food and Drug Administration, US Department of Agriculture Food Safety and Inspection Service; 2017 [cited 2020 May 4]. https://www.cdc.gov/foodsafety/ifsac/annual-reports.html

[32] Interagency Food Safety Analytics Collaboration. Foodborne illness source attribution estimates for 2017 for *Salmonella, Escherichia coli* O157, *Listeria monocytogenes*, and *Campylobacter* using multi-year outbreak surveillance data, United States. Atlanta and Washington: Centers for Disease Control and Prevention, US Food and Drug Administration, US Department of Agriculture Food Safety and Inspection Service; 2019 [cited 2020 May 4]. https://www.cdc.gov/foodsafety/ifsac/annual-reports.html

