# Supplementary material for: Recency-Weighted Statistical Modeling Approach to Attribute Illnesses Caused by 4 Pathogens to Food Sources Using Outbreak Data, United States
Source: Emerg Infect Dis. 2021 Jan;27(1):214–22. doi: 10.3201/eid2701.203832 (PMC7774545; doi:10.3201/eid2701.203832)
Supplement: Appendix — Additional information about recency-weighted statistical modeling approach to attribute illnesses caused by 4 pathogens to food sources using outbreak data, United States. [file 20-3832-Techapp-s1.pdf]

Article DOI: https://doi.org/10.3201/eid2701.203832

Recency-Weighted Statistical Modeling Approach to Attribute Illnesses Caused by 4 Pathogens to Food Sources Using Outbreak Data, United States

Appendix

IFSAC Food Categorization Scheme

Appendix Figure 1 shows the categorization scheme used to classify foods implicated in outbreaks. The scheme and the associated methodology used to assign outbreaks to food categories based on implicated foods and ingredients, as well as examples of foods assigned to each category, are described in Richardson et al. (*1*).

Description of Data Set

This section provides a general description of the data used in the source attribution model described in this article.

We extracted data on 16,584 foodborne disease outbreaks reported in the 15 years from 1998 through 2012 from CDC's Foodborne Disease Outbreak Surveillance System (FDOSS) (https://www.cdc.gov/foodsafety/fdoss) (*2*). These data were extracted on December 18, 2013.

Appendix Figure 2 shows the stages of data preparation. Specifically, it shows the number of outbreaks excluded from the analysis at each stage (shaded boxes) and the number remaining (unshaded boxes).

First, we excluded outbreaks that occurred in outlying U.S. territories (e.g., Puerto Rico). Next, we excluded the 83% of remaining outbreaks not caused by the 4 priority pathogens: nontyphoidal *Salmonella*, *Escherichia coli* O157 (namely, *E. coli* O157:H7 and *E. coli* O157:NM), *Listeria monocytogenes*, and *Campylobacter* spp. Of these 2,732 outbreaks, we further excluded 77 outbreaks caused by multiple pathogens.

Of the resulting 2,655 outbreaks caused by one of the 4 priority pathogens as the single etiology, we excluded 38% (n = 1,014) because investigators did not identify an implicated food and 26% (n = 689) because the implicated food(s) could not be assigned to a single food category. Implicated foods could not be assigned to a single food category because the identified food was complex (composed of ingredients belonging to more than one food category) (n = 448); or foods from more than one food category were implicated or suspected (i.e., multiple foods) (n = 142); or the food was too vaguely described to be assigned to any category (e.g., “buffet,” “appetizer”) (n = 50); or the food was too vaguely described to be assigned to the specific food categories used in the analysis (e.g., could only be assigned to “Produce” or “Meat-Poultry”) (n = 49).

We focus on single-pathogen single-food category outbreaks because the appropriate categorization of both pathogens and foods is known. A method is in development for assigning to multiple food categories those outbreaks due to complex foods for which the implicated ingredient was unknown. The previously published approach could not be applied to our data series without substantial revisions (*3*). This approach used “recipes” developed using internet searches and these recipes would need to be updated to reflect current online recipes and to incorporate changes to food categories (*4*).

Thus, our final dataset for analysis included outbreaks caused by a single pathogen that could be assigned to one of 22 specific food categories; these were 952 (36%) of the 2,655 single etiology outbreaks. The pathogen with the most outbreaks in the resulting data was *Salmonella* (n = 597), the predominant serotype of which was Enteritidis (n = 184) (Appendix Table 1). There were 170 outbreaks caused by *E. coli* O157, 24 by *L. monocytogenes*, and 161 by *Campylobacter* (Appendix Figure 2).

As part of preliminary analyses, we assessed the quality of information on etiology status. In FDOSS, an outbreak must have at least 2 ill persons (*2*). For *Salmonella*, *E. coli* O157, and *Campylobacter*, outbreaks with “confirmed” etiology are defined as those in which the outbreak strain was isolated from at least 2 patients or from epidemiologically implicated food; “confirmed” outbreaks of *L. monocytogenes* infections must have 1 person with the outbreak strain isolated from a normally sterile site (*5*). (Cases of listeriosis can also be diagnosed based on symptoms and culture of products of conception, which are not sterile.) The etiology of an

outbreak not meeting these conditions is considered to be “suspected.” Of the 2,732 outbreaks associated with the 4 priority pathogens, 90% (2,462) were coded as having confirmed etiology. We found that 12% of outbreaks coded as having confirmed etiology did not have sufficient data to fulfill the confirmed etiology definition, but also found that over 95% of outbreaks coded as having suspected etiology had at least one laboratory-confirmed illness. Outbreaks occurring early in the study period were more likely to have insufficient data to confirm an etiology.

We decided to include outbreaks with either confirmed or suspected etiology status in the analysis so as not to lose information associated with those outbreaks, following the decision made in Painter et al. (*3*). We also conducted a sensitivity analysis on this decision, as described elsewhere in this appendix.

Of the 952 outbreaks in the data used to estimate attribution percentages, 83 (8.7%) did not have a confirmed etiology, including 8.9% (n = 53) of *Salmonella* outbreaks, 4.1% (n = 7) of *E. coli* O157 outbreaks, 12.5% (n = 3) of *L. monocytogenes* outbreaks, and 12.4% (n = 20) of *Campylobacter* outbreaks.

NORS includes 3 variables related to outbreak size: the number of lab-confirmed primary cases (ConfirmedPrimary), the number of additional illnesses that were not laboratory confirmed (ProbablePrimary), and the total of both confirmed and probable illnesses (EstimatedPrimary). In our attribution estimates, we use the estimated total illnesses as our measure of outbreak size.

Statistical Model Development

This section provides additional details about the models used to estimate the food sources of illnesses. Specifically, it describes development of pathogen-specific statistical models of outbreak size, and the approach used to weight recent outbreaks more heavily than older outbreaks.

Analysis of variance models

Log-transforming outbreak size resulted in relatively normally distributed outbreak illness numbers that could be modeled using straightforward analysis of variance (Te) modeling techniques. We explored several modeling approaches, including analysis of covariance (ANCOVA), generalized linear models, and least absolute shrinkage and selection operator (LASSO) models, among others. We decided to use ANOVA based on structural simplicity and

interpretability, and because our data were not sufficient to credibly describe the complexity of interactions between the epidemiologic characteristics of reported outbreaks.

We developed pathogen-specific models because we did not want to smooth over differences in outbreak size by pathogen, as this variation likely results from epidemiologic factors, not random variation. We found outbreaks caused by different *Salmonella* serotypes varied in foods implicated and other epidemiologic factors. In particular, serotype Enteritidis outbreaks had some distinct patterns. Thus, we decided to model serotype Enteritidis separately from all the other serotypes for estimating outbreak size; when we calculate attribution percentages in a subsequent stage, we do so after summing the 2 sets of model estimates.

Based on preliminary modeling analysis and considerations of epidemiologic importance, we included 3 variables as the predictors of outbreak size in all 5 pathogen-specific models: food category, the type of location at which the food was prepared, and whether outbreak exposures occurred in a single state or in multiple states. Each outbreak was assigned to 1 of 17 food categories, as described previously. The food preparation location variable used in the model included 5 categories, based on 24 individual location types identified in outbreak reports, as shown in Appendix Table 2. We reduced the number of categories to 5 to address the relatively sparse data across most locations other than restaurant or private home. A dichotomous variable was used to indicate whether exposures occurred in multiple states or a single state.

We desired a model that was portable in that it could be similarly described across the 4 etiologies included in the study and expandable to additional pathogens. Summary measures for model fit are shown in Appendix Table 3, including traditional lack-of-fit, R-squared, overall model significance, and significance of each predictor. Appendix Table 3 also shows, in the last 3 columns, variance explained via random forest decomposition using identical predictors. Appendix Figure 3 compares the number of reported illnesses with the number of model-estimated illnesses and shows that, as expected, our ANOVA models reduce variation in outbreak size and the influence of very large outbreaks.

Recency weighting

The decision to down-weight older data was made because recent outbreaks are likely to be more representative of current foodborne illness attribution than older outbreaks. Changes in attributable risk may result from changes over time in food consumption patterns, food

production and processing practices, food safety activities, regulatory interventions, and other factors.

This decision is supported by characteristics of the underlying data. Appendix Figure 4 presents a heat map with the number of outbreaks by pathogen and food category over time. White cells indicate no outbreaks due to that pathogen-food category pair in that year, with color from pale orange to red indicating between 1 and 25 outbreaks in that year. Appendix Figure 4 illustrates the variability in data sparseness across many pathogen-food categories.

We examined the impacts of excluding older data by estimating attribution for 3, 5-year time frames: 1998–2002, 2003–2007, and 2008–2012. Appendix Figure 5 displays the estimated attribution percentages (y-axis) by food category (lines) and timeframe (x-axis). There are notable differences across these timeframes. The variability of underlying data leads to instability in estimated percentages based on short time windows. Excluding older data entirely results in estimates of zero attribution for some categories with known nonzero risk.

Based on this and other analyses, we decided that outbreaks older than 5 years should be included in estimates of attribution but down-weighted to increase the relative influence of more recent outbreaks on attribution estimates.

As described in the article, we determined that the most appropriate approach would be to use an exponential decay function to define the recency-weighting multiplier *w* for an outbreak in year *y*, as a function of decay parameter *a*:

$$w_y = \begin{cases} a^{2008-y}, y < 2008 \\ 1, y \geq 2008 \end{cases}$$

We evaluated various options for the decay parameter *a* and the resulting weighting factor by year, as shown in Appendix Figure 6. Our preference was for more than half of the information in our estimates to come from the most recent 5 year period, and a small amount – around 5% – from data older than 10 years. Because the distribution of outbreak illnesses is not constant over time or by pathogen (as shown in Appendix Figure 4), we selected a decay parameter that best met our preferences for all pathogens. As shown in Appendix Table 4, with a decay parameter value of 0.7142 (5/7), 67% of the total down-weighted model-estimated outbreak illnesses used in the attribution calculation were from outbreaks that occurred during

the most recent 5-year period (2008–2012), with ≈28% from the middle 5-year period, and 5% from the oldest 5-year period.

Sensitivity Analyses

This appendix describes sensitivity analyses conducted to assess the robustness of our attribution estimates. We compare our model-based estimates to those derived used in prior studies and explore sensitivities to modeling decisions and underlying data.

Sensitivity to Use of Statistical Modeling and Recency-weighting

Prior estimates of foodborne illness source attribution based on outbreaks have summed the raw number of reported outbreaks or outbreak illnesses associated with a given pathogen-food category pair and divided this by the total number of outbreaks or outbreak illnesses associated with that pathogen (*3*,*6*). By contrast, our estimates are based on statistical modeling of log-transformed outbreak size, with exponential down-weighting of older outbreaks (we refer to these as our "baseline" estimates).

Appendix Figure 7 compares our model-based attribution percentages (with and without down-weighting of older outbreaks) to those based on raw numbers of reported outbreaks and outbreak illnesses. Attribution percentages are shown in log scale to better highlight differences. Differences reflect dependencies between multinomial estimates; because attribution percentages sum to 100%, a downward shift in the percentage for one food category results in higher percentages elsewhere.

Appendix Figure 7 illustrates 3 points. First, it shows the range of estimates using the methods in the published literature, namely that there are notable differences between attribution estimates based on numbers of reported outbreaks (purple lines) and numbers of outbreak illnesses (pink lines). Pathogen-food category pairs with the largest differences are *Salmonella* in Seeded Vegetables and Chicken, *E. coli* O157 in Fruits and Sprouts, *L. monocytogenes* in Fruits and Turkey, and *Campylobacter* in Chicken and Other Seafood. These differences reflect the outbreak size variation across food categories, as well as the impact of very large outbreaks.

Second, Appendix Figure 7 also shows that although our attribution estimates are generally similar to estimates based on the approaches used in the published literature, there are some differences. For *Salmonella*, the Seeded Vegetables category has the highest attribution

percentage in our baseline estimates, whereas the Eggs category has the highest percentage based on numbers of reported outbreaks (purple) or outbreak illnesses (pink). Chicken also has higher attribution percentages based on counts of reported outbreaks or outbreak illnesses, compared to the baseline, whereas the Sprouts category has a lower estimate. For *E. coli* O157, baseline estimates for the Vegetable Row Crops category are higher than those based on reported outbreaks or outbreak illnesses. For *L. monocytogenes*, our baseline estimates are closest to those based on counts of reported outbreak illnesses, though the baseline estimate for Turkey is lower than those based on counts of reported outbreaks or outbreak illnesses, and the baseline estimate for Fruits is respectively higher. There are fewer differences in *Campylobacter* estimates, though the Seeded Vegetables category has a notably higher attribution percentage when based on model-estimated illnesses.

Lastly, Appendix Figure 7 shows that eliminating recency-weighting affects attribution percentages, though not drastically. For *Salmonella*, attribution percentages calculated without recency-weighting (green) are higher for Chicken and Eggs, and lower for Seeded Vegetables and Vegetable Row Crops, compared to baseline estimates with recency-weighting (red). For *E. coli* O157, estimates for Dairy and Vegetable Row Crops are marginally lower than the baseline without recency-weighting; estimates for Sprouts and Fruits are marginally higher. For *L. monocytogenes*, removing recency-weighting results in lower estimates for Fruits and higher estimates for Turkey, reflecting the impact of the large cantaloupe outbreak in 2011, its recency, and the fact that there were not any *L. monocytogenes* outbreaks traced to turkey luncheon meat between 2005 and 2012 (Appendix Figure 4). For *Campylobacter*, removing recency-weighting results in a higher estimate for Other Produce, reflecting the impact of a large 2002 prison outbreak associated with potatoes (the only outbreak in this pathogen-food category (Appendix Figure 4).

Sensitivity to ANOVA Model Specifications

As noted in the text and other appendices, we conducted exploratory analyses to determine which predictors should be included in the pathogen-specific ANOVA models. The final 3-predictor model specifications were based both on epidemiologic reasoning and our findings that these variables were statistically significant predictors of outbreak size.

We conducted sensitivity analyses around the final model specification by estimating attribution percentages using 3 alternative ANOVA models: one without the dichotomous multi-state variable, one without the categorical preparation location variable, and one without either. The results (Appendix Figure 8) show that our model is robust to model specification decisions in comparison with the baseline model specification.

Sensitivity to Etiology Status

As described previously, we included outbreaks with "suspected" etiology in addition to those with laboratory-confirmed isolates from patients or food in the analysis. Those without confirmed etiology comprise ≈9% of the outbreaks used in the analysis, though of these, most had at least one laboratory-confirmed illness. We conducted a sensitivity analysis around this decision. Appendix Figure 9 presents our baseline attribution estimates and 90% credibility intervals alongside estimates based on data excluding the 83 outbreaks with suspected etiology. Appendix Figure 9 shows that for all but a few pathogen-food category pairs, the differences in point estimates are minimal, though credibility intervals are wider when outbreaks of suspected etiology are excluded.

Sensitivity to Influential Outbreaks

We conducted a series of analyses to identify which outbreaks are most influential on our attribution estimates and to assess model sensitivity to these outbreaks. This was done in part to ascertain the extent to which our estimates were sensitive to very large outbreaks, though because our estimates are based on a 3-parameter statistical model of log-transformed outbreak size, with recency-weighting, we needed a systematic approach to identify influential outbreaks.

The first step was to define an influence metric for each outbreak based on the aggregate difference in attribution estimates when that outbreak was excluded from the analysis. That is, for each of 952 outbreaks, we estimated attribution percentages without that single outbreak. We defined an "influence metric" as the sum of mean differences squared across all pathogen-food category pairs between the baseline estimate and the estimate without that outbreak; the attribution percentages change only for the pathogen for which an outbreak was excluded. We then calculated the overall "influence rank" for each outbreak based on the rank order of the "influence metric."

Appendix Figure 10 presents, for each pathogen, the calculated influence metric for each outbreak, in descending order. These plots show that most outbreaks have influence metrics at or very close to zero, but a small number do have measurable influence metrics. Appendix Figure 10 shows that the 10 outbreaks most influential on attribution estimates were caused by *L. monocytogenes* and *Campylobacter*. Appendix Table 5 provides details for the 5 outbreaks most influential on attribution estimates for each pathogen. Although the plots in Appendix Figure 10 show that some outbreaks have large influence metric values, the actual impacts of these individual outbreaks on attribution estimates is minimal.

Appendix Figure 11 presents estimates for scenarios in which each of the 5 outbreaks most influential on attribution estimates (from Appendix Table 5) for each pathogen was excluded one at a time. These scenarios are shown alongside the baseline attribution percentages. Appendix Figure 11 shows that for all but the single most influential outbreak (*L. monocytogenes* in cantaloupe), the exclusion of any single outbreak results in negligible differences in attribution estimates, and no differences in the rank order of food categories. We therefore concluded that our model is robust to all but the most extreme outliers, and that only our estimates for *L. monocytogenes* are sensitive to the impact of individual outbreaks.

References

1. Richardson LC, Bazaco MC, Parker CC, Dewey-Mattia D, Golden N, Jones K, et al. An updated scheme for categorizing foods implicated in foodborne disease outbreaks: a tri-agency collaboration. Foodborne Pathog Dis. 2017;14:701–10. PubMed https://doi.org/10.1089/fpd.2017.2324

2. Gould LH, Walsh KA, Vieira AR, Herman K, Williams IT, Hall AJ, et al.; Centers for Disease Control and Prevention. Surveillance for foodborne disease outbreaks—United States, 1998–2008. MMWR Surveill Summ. 2013;62:1–34. PubMed

3. Painter JA, Hoekstra RM, Ayers T, Tauxe RV, Braden CR, Angulo FJ, et al. Attribution of foodborne illnesses, hospitalizations, and deaths to food commodities by using outbreak data, United States, 1998-2008. Emerg Infect Dis. 2013;19:407–15. PubMed https://doi.org/10.3201/eid1903.111866

4. Painter JA, Ayers T, Woodruff R, Blanton E, Perez N, Hoekstra RM, et al. Recipes for foodborne outbreaks: a scheme for categorizing and grouping implicated foods. Foodborne Pathog Dis. 2009;6:1259–64. PubMed https://doi.org/10.1089/fpd.2009.0350

5. Centers for Disease Control and Prevention. Guide to confirming an etiology in foodborne disease outbreak. 2015 [cited 2018 July 12]. https://www.cdc.gov/foodsafety/outbreaks/investigating-outbreaks/confirming_diagnosis.html

6. Batz MB, Hoffmann S, Morris JG Jr. Ranking the disease burden of 14 pathogens in food sources in the United States using attribution data from outbreak investigations and expert elicitation. J Food Prot. 2012;75:1278–91. PubMed https://doi.org/10.4315/0362-028X.JFP-11-418

Appendix Table 1. Number of nontyphoidal *Salmonella* outbreaks and outbreak illnesses due to a single food category — Foodborne Disease Outbreak Surveillance System, United States, 1998–2012*

Food category	Serotype Enteritidis	Other serovars	All serovars
Beef	9 (157)	38 (1,316)	47 (1,473)
Pork	8 (167)	43 (931)	51 (1,098)
Chicken	26 (580)	88 (2,068)	114 (2,648)
Turkey	7 (420)	42 (888)	49 (1,308)
Other meat, poultry	1 (13)	5 (71)	6 (84)
Game	1 (3)	1 (5)	2 (8)
Dairy	1 (39)	23 (754)	24 (793)
Eggs	113 (4,895)	27 (350)	140 (5,245)
Fish	2 (82)	10 (204)	12 (286)
Other seafood	2 (26)	2 (10)	4 (36)
Grains, beans	0 (0)	7 (268)	7 (268)
Oils, sugars	0 (0)	0 (0)	0 (0)
Fruits	5 (240)	41 (2,270)	46 (2,510)
Seeded vegetables	1 (85)	33 (3,916)	34 (4,001)
Sprouts	5 (174)	28 (1,092)	33 (1,266)
Vegetable row crops	1 (14)	9 (398)	10 (412)
Other produce	2 (45)	16 (1,878)	18 (1,923)
Total	184 (6,940)	413 (16,419)	597 (23,359)

*Number of outbreak-associated illnesses in parentheses. Nontyphoidal *Salmonella* is divided into *S. enterica* ser. Enteritidis and other serovars.

Appendix Table 2. Types of food preparation locations as defined in reported outbreak data and aggregated categories and outbreak counts used in statistical models of outbreak size

Preparation locations identified in outbreak line listings	Categories used ANOVA model
Restaurant – "Fast-food"<br>Restaurant – other/unknown type<br>Restaurant – Sit-down dining	Restaurant
Restaurant or deli	Private Home
Private Home<br>Banquet facility<br>Caterer<br>Caterer (food prepared off-site)<br>Fair, festival, other temporary or mobile service<br>Picnic<br>Camp<br>Day care center<br>Hospital<br>Nursing home, assisted living, home care<br>School<br>Commercial product, no further preparation<br>Grocery store<br>Church, temple, or other religious location<br>Prison, jail<br>Other	Other
Contaminated food imported into U.S.<br>Unknown or Undetermined	Unknown
No Data	Multiple

Appendix Table 3. Summary measures for pathogen-specific ANOVA models of outbreak size for *Salmonella*, *E. coli* O157, *L. monocytogenes*, and *Campylobacter*, based on analysis of single pathogen, single food category outbreaks – Foodborne Disease Outbreak Surveillance System, 1998–2012

	Lack-of-fit		R-squared		Significance (P-value)				Explained Variance		
Pathogen	**Degrees of Freedom**	**P-value**	**Max**	**Model**	**Overall Model**	**Food Category**	**Multi-state**	**Prep. Location**	**Food Category**	**Multi-state**	**Prep. Location**
Salmonella											
Enteritidis	21	0.88	0.32	0.20	<0.001	0.40	<0.001	<0.001	0.22	0.35	0.42
Other serotypes	76	0.45	0.47	0.33	<0.001	<0.05	<0.001	<0.001	0.28	0.54	0.18
E. coli O157	24	0.25	0.45	0.32	<0.001	<0.05	<0.001	0.26	0.27	0.58	0.15
L. monocytogenes	4	0.14	0.90	0.65	<0.05	0.09	0.15	0.55	0.55	0.33	0.12
Campylobacter	14	0.23	0.28	0.14	0.05	0.15	0.70	0.09	0.61	0.00	0.39

Appendix Table 4. Proportion of outbreak information in attribution estimates under alternative recency-weighting decay parameters

	Decay Parameter			
Years of Data	**0.20**	**0.50**	**0.71***	**0.80**
1998–2002	0%	<1%	5%	10%
2003–2007	5%	16%	28%	31%
2008–2012	95%	83%	67%	58%

* Decay parameter selected for baseline model.

Appendix Table 5. The 5 most influential outbreaks on attribution estimates, for *Salmonella*, *E. coli* O157, *L. monocytogenes*, and *Campylobacter*, based on analysis of single pathogen, single food category outbreaks — Foodborne Disease Outbreak Surveillance System, 1998–2012*

Overall Influence Rank	Food Category	Year	Food Item(s) Implicated	Preparation setting	Multistate	No. Illnesses	Log (No. Illnesses)	Influence Metric
Salmonella								
17	Eggs	2010	shell egg, other (egg)	Multiple	Yes	1939	7.6	7.4
22	Seeded veg.	2008	jalapeno/serrano peppers, tomato	Unknown	Yes	1500	7.3	5.7
30	Other Produce	2008	peanut butter, peanut paste	Unknown	Yes	714	6.6	4.7
32	Seeded veg.	2009	ground pepper (in salami)	Other	Yes	272	5.6	4.1
40	Other Produce	2006	peanut butter	Other	Yes	715	6.6	2.9
E. coli O157								
12	Veg. Row Crops	2006	spinach	Multiple	Yes	238	5.5	9.7
14	Veg. Row Crops	2008	iceberg lettuce	Unknown	Yes	74	4.3	9.2
16	Veg. Row Crops	2011	romaine lettuce	Multiple	Yes	60	4.1	8.2
18	Veg. Row Crops	2012	romaine lettuce	Unknown	Yes	52	4.0	7.2
20	Fruits	2000	watermelon	Restaurant	No	736	6.6	6.8
L. monocytogenes								
1	Fruits	2011	cantaloupe	Private Home	Yes	147	5.0	3560.4
2	Dairy	2012	ricotta salata cheese	Multiple	Yes	23	3.1	51.4
3	Sprouts	2008	sprouts	Multiple	Yes	20	3.0	41.0
5	Dairy	2009	Mexican-Style Cheese	Private Home	Yes	18	2.9	25.8
7	Dairy	2011	blue-veined cheese, unpasteurized	Other	Yes	15	2.7	21.3
Campylobacter								
4	Seeded veg.	2008	green peas	Other	No	104	4.6	32.7
6	Other Seafood	2008	raw and steamed clams	Multiple	No	268	5.6	22.3
10	Pork	2008	pork	Other	No	27	3.3	11.7
11	Other Seafood	2010	raw clams	Other	No	68	4.2	11.4
15	Other Seafood	1998	oysters	Private Home	No	2	0.7	8.6

*Overall influence rank is based on the rank order of outbreaks when sorted by the influence metric, shown in the last column. The influence metric is defined as the sum of mean differences squared across all pathogen-food category pairs between the baseline estimate and an attribution estimate with that outbreak excluded.

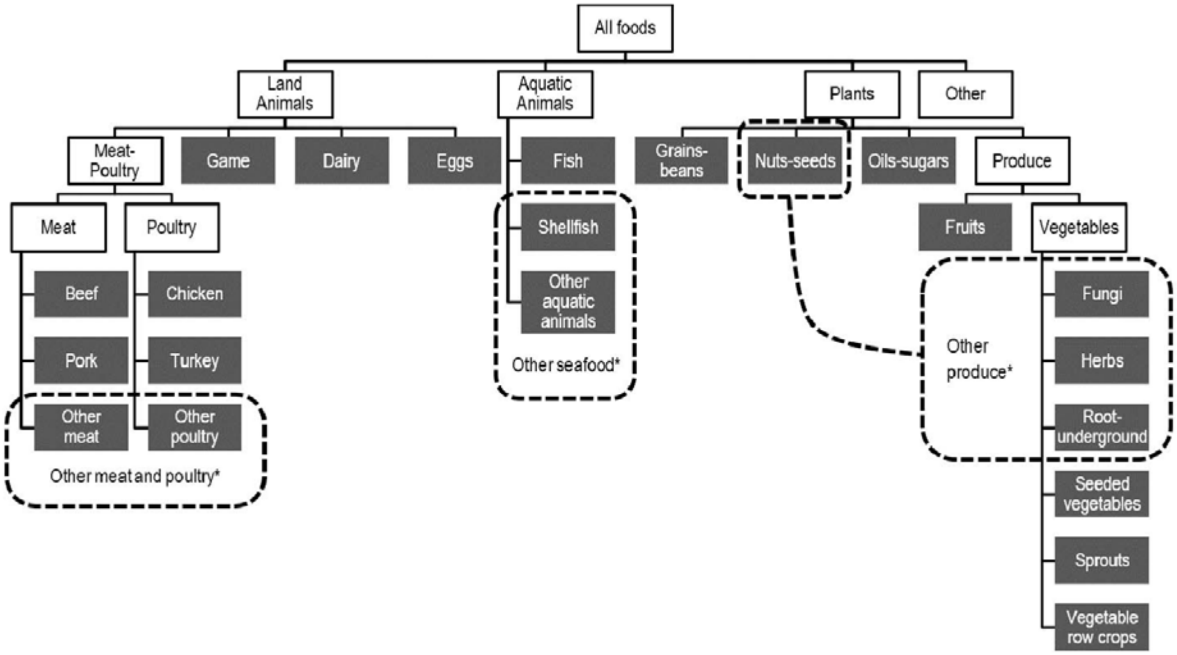

Appendix Figure 1. Hierarchical scheme used to categorize foods implicated in foodborne disease outbreaks. Outbreaks were assigned to one of 22 food categories (dark gray boxes) in the IFSAC food categorization scheme. Due to sparse data, 8 of these food categories were aggregated into 3 combined categories as indicated by the dashed-line boxes, resulting in the 17 food categories used in this analysis. “Other meat and poultry” includes animal species other than beef, pork, chicken and turkey.

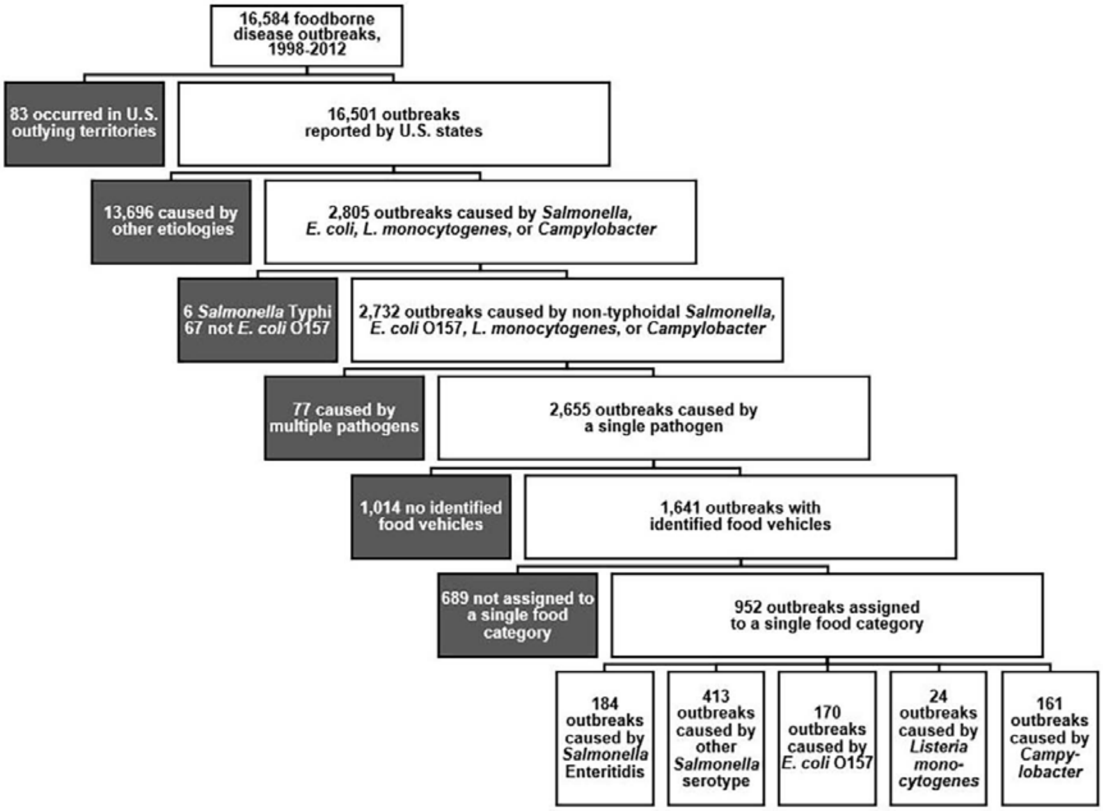

Appendix Figure 2. Data tree showing the number of outbreaks included and excluded in analysis – Foodborne Disease Outbreak Surveillance System, United States, 1998–2012.

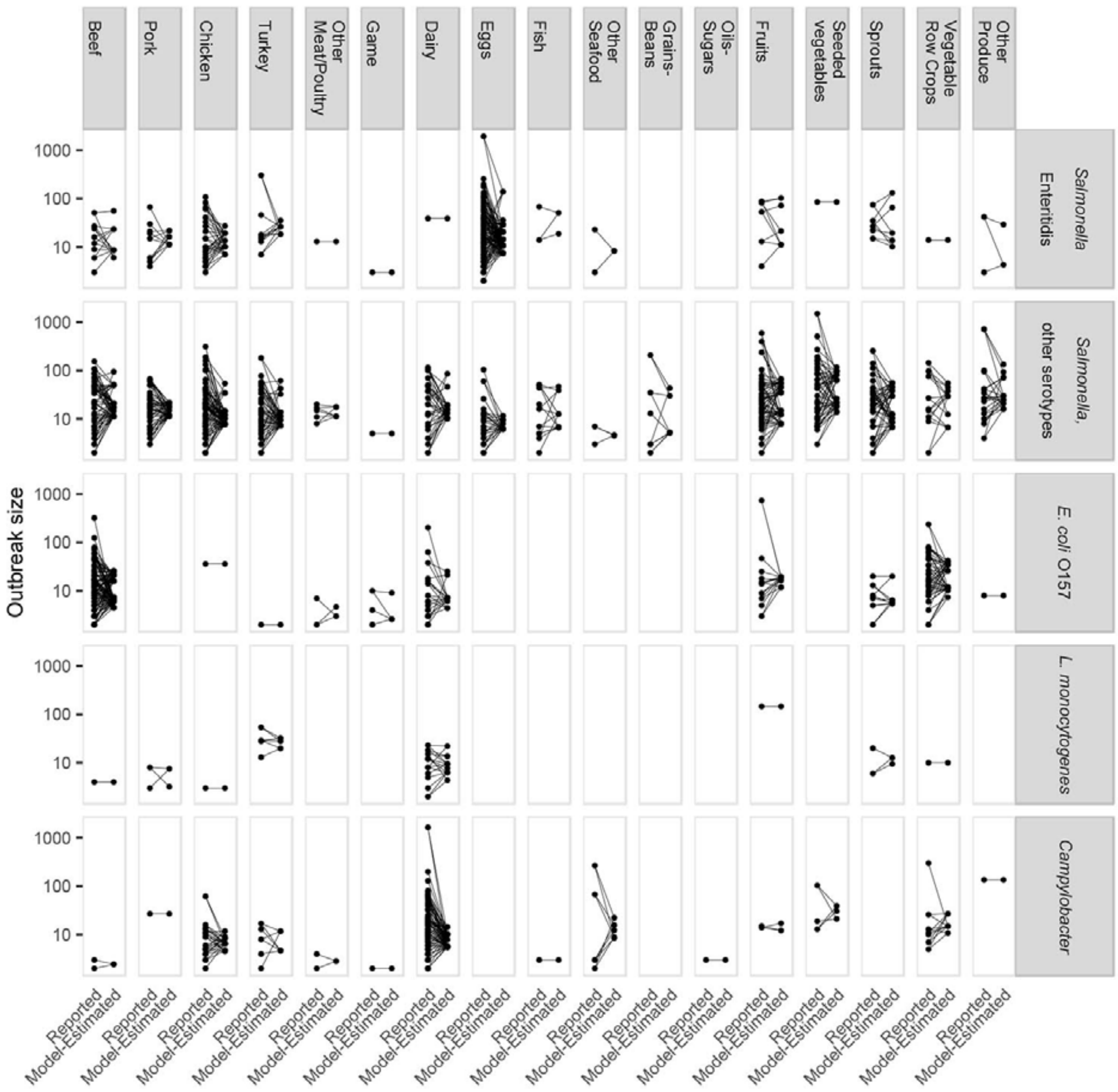

Appendix Figure 3. Comparison of reported and model-estimated number of illnesses per outbreak, by food category, for *Salmonella* Enteriritidis, other *Salmonella* serotypes, *E. coli* O157, *L. monocytogenes*, and *Campylobacter*, based on analysis of single pathogen, single food category outbreaks – Foodborne Disease Outbreak Surveillance System, 1998–2012. Each line in each panel represents a single outbreak, with a line connecting the number of reported illnesses (dot on left) with the number of model-estimated illnesses (dot on right), both presented on the same log-scale. The resulting sideways triangular shape of the combined lines in each panel illustrates the reduced variation achieved by modeling.

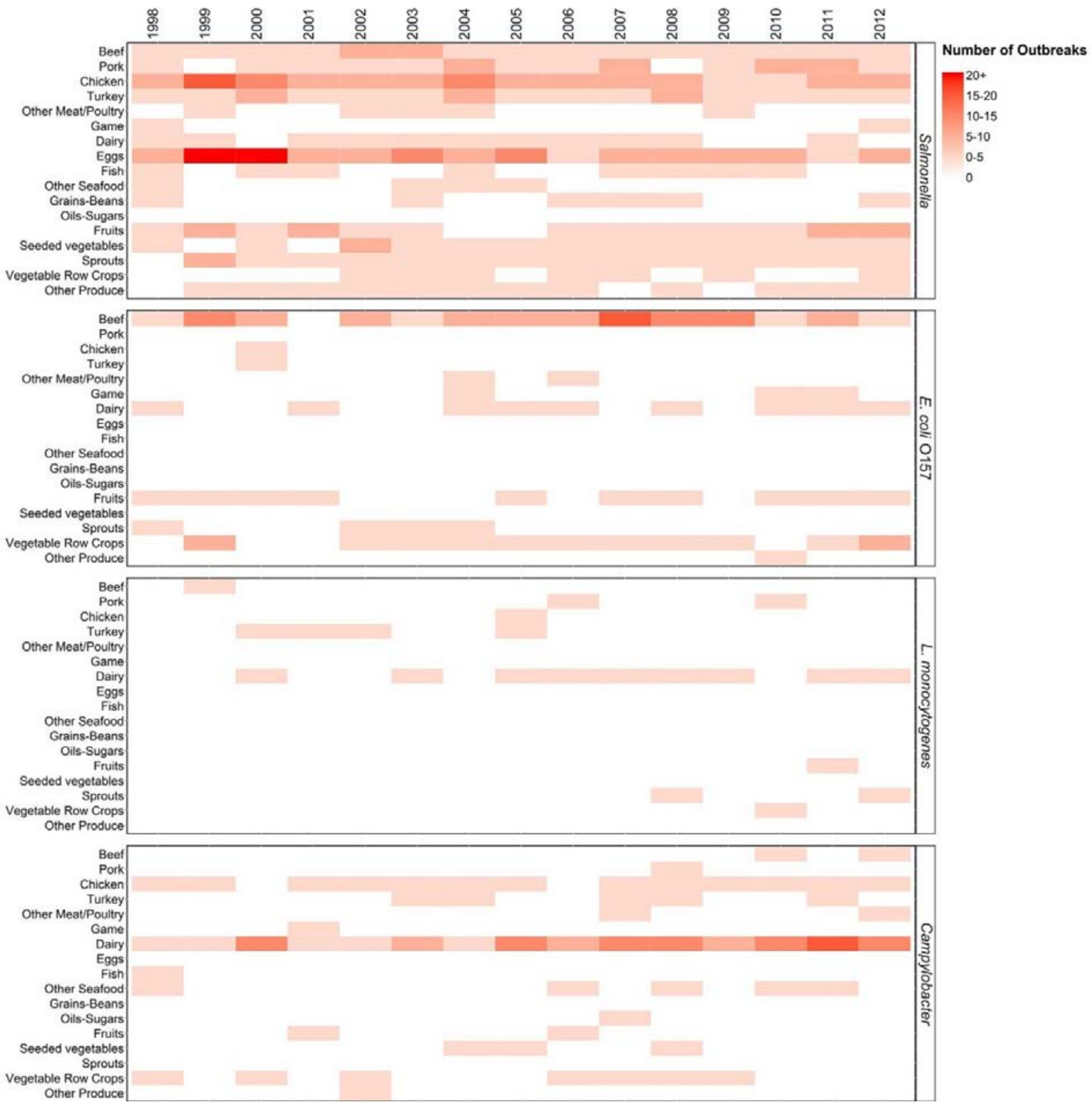

Appendix Figure 4. Number of reported outbreaks caused by a single pathogen and due to a single food category, by food category and year, for *Salmonella*, *E. coli* O157, *L. monocytogenes*, and *Campylobacter* – Foodborne Disease Outbreak Surveillance System, United States, 1998–2012.

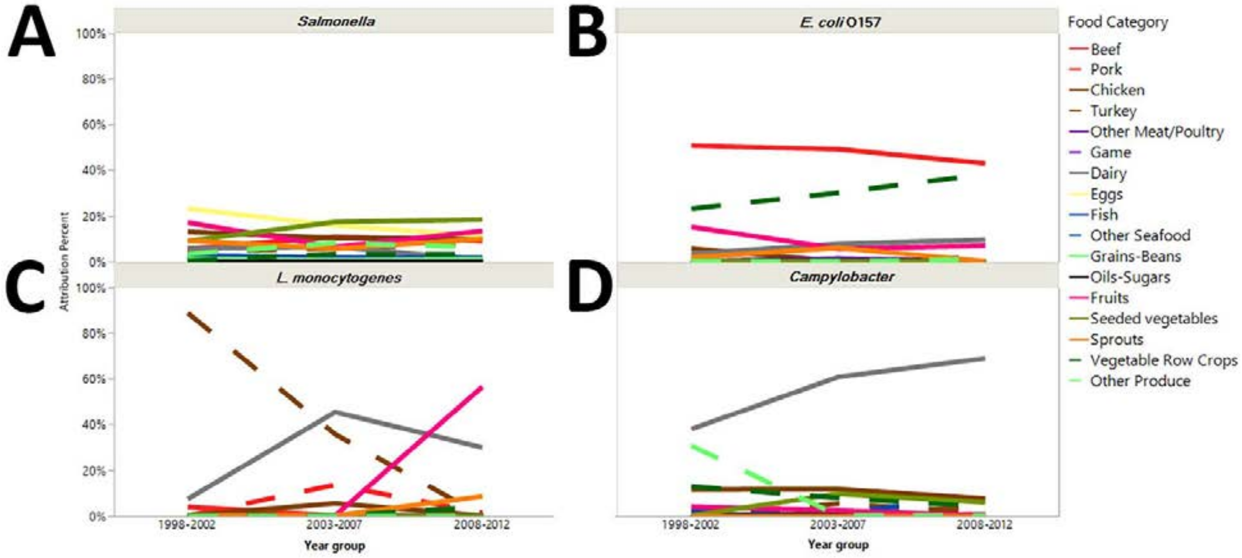

Appendix Figure 5. Estimated percentages of illnesses caused by *Salmonella*, *E. coli* O157, *L. monocytogenes*, and *Campylobacter* attributed to food categories for 5-year windows, based on analysis of single pathogen, single food category outbreaks – Foodborne Disease Outbreak Surveillance System, 1998–2012. Estimates calculated using ANOVA model-estimated outbreak illnesses for single pathogen, single food category outbreaks from 1998–2012, with down-weighting of outbreaks from 1998–2007.

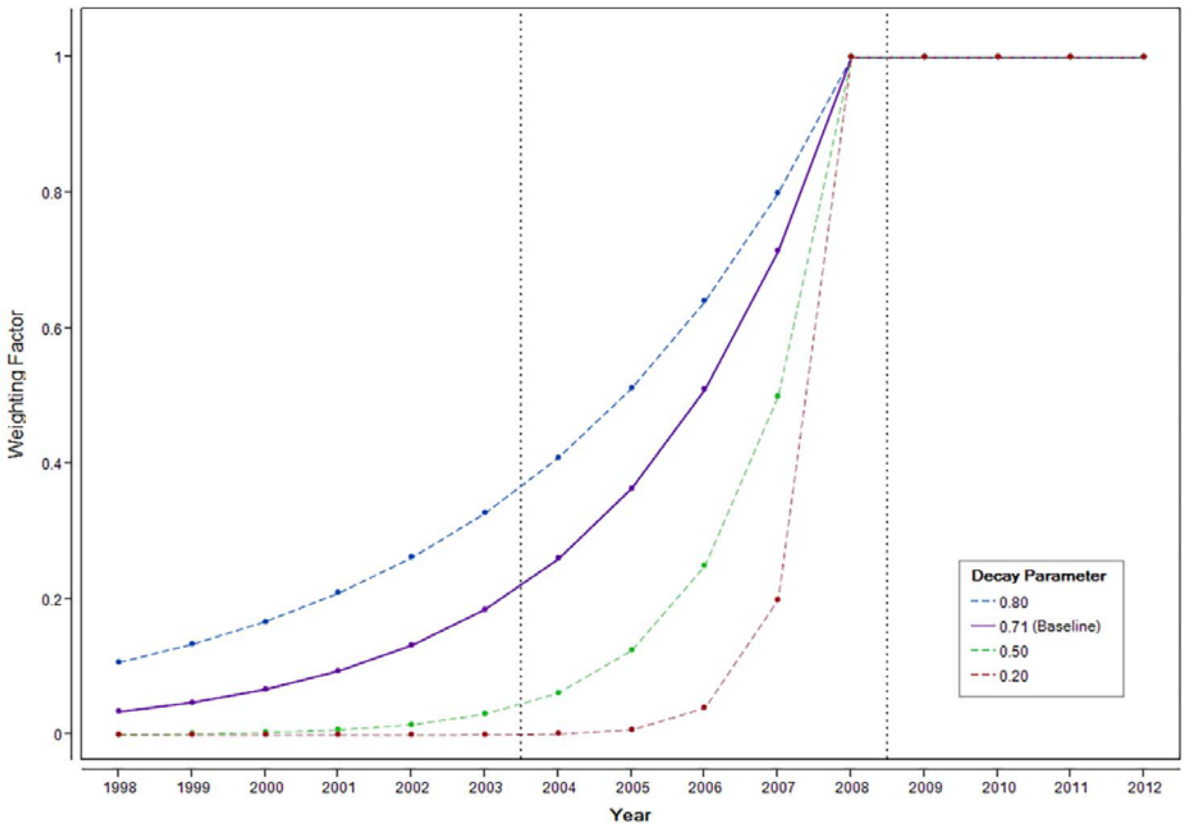

Appendix Figure 6. Comparison of multiplicative weighting factors evaluated to recency-weight model-estimated outbreak illnesses, by year and decay parameter.

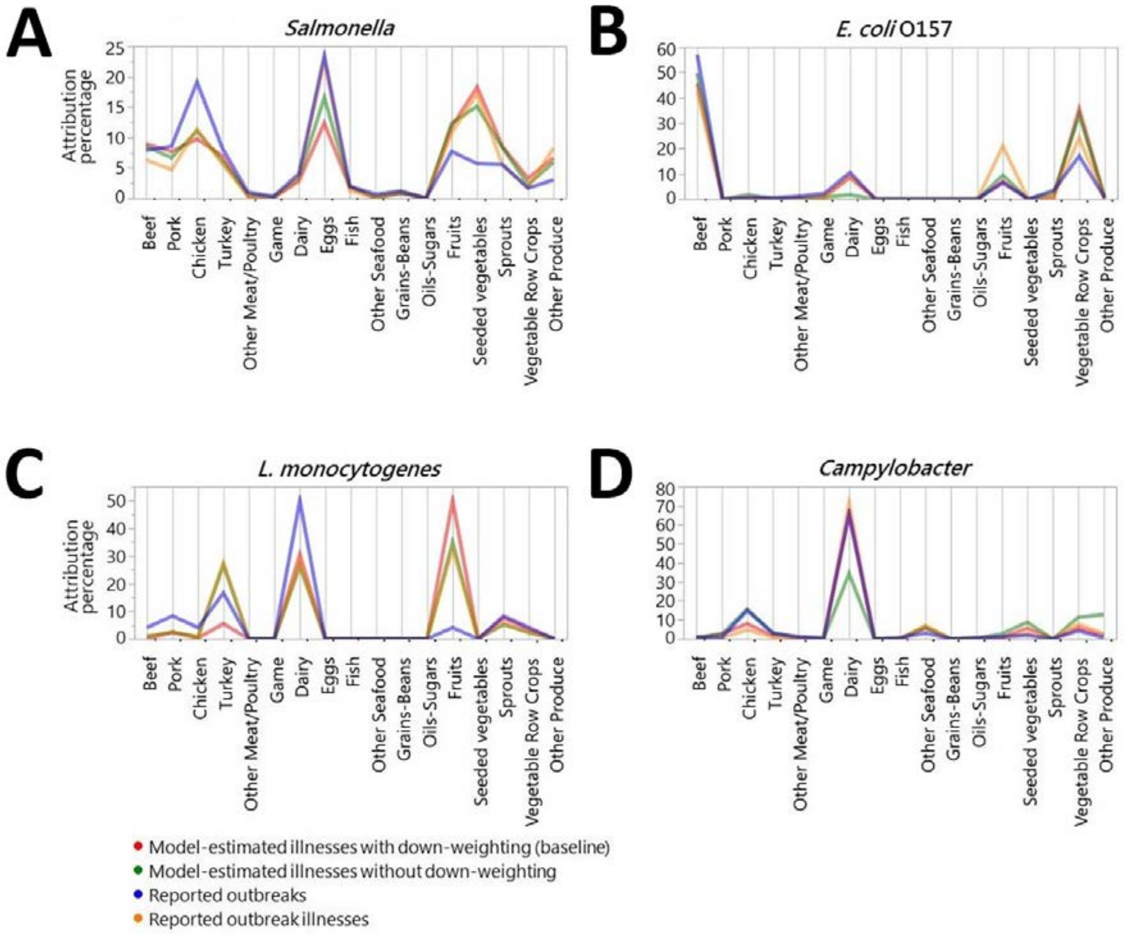

Appendix Figure 7. Comparison of measures used to calculate estimated percentages of illnesses attributed to 17 food categories for *Salmonella*, *E. coli* O157, *L. monocytogenes*, and *Campylobacter*, based on analysis of single pathogen, single food category outbreaks – Foodborne Disease Outbreak Surveillance System, 1998–2012. Each panel shows baseline estimated attribution percentages based on numbers of model-estimated illnesses with down-weighting of older outbreaks (in red). This is compared to attribution percentages based on model-estimated illnesses without down-weighting (green), and to attribution percentages calculated without any statistical modeling – namely, based on the number of reported outbreaks (purple) and number of reported outbreak illnesses (orange).

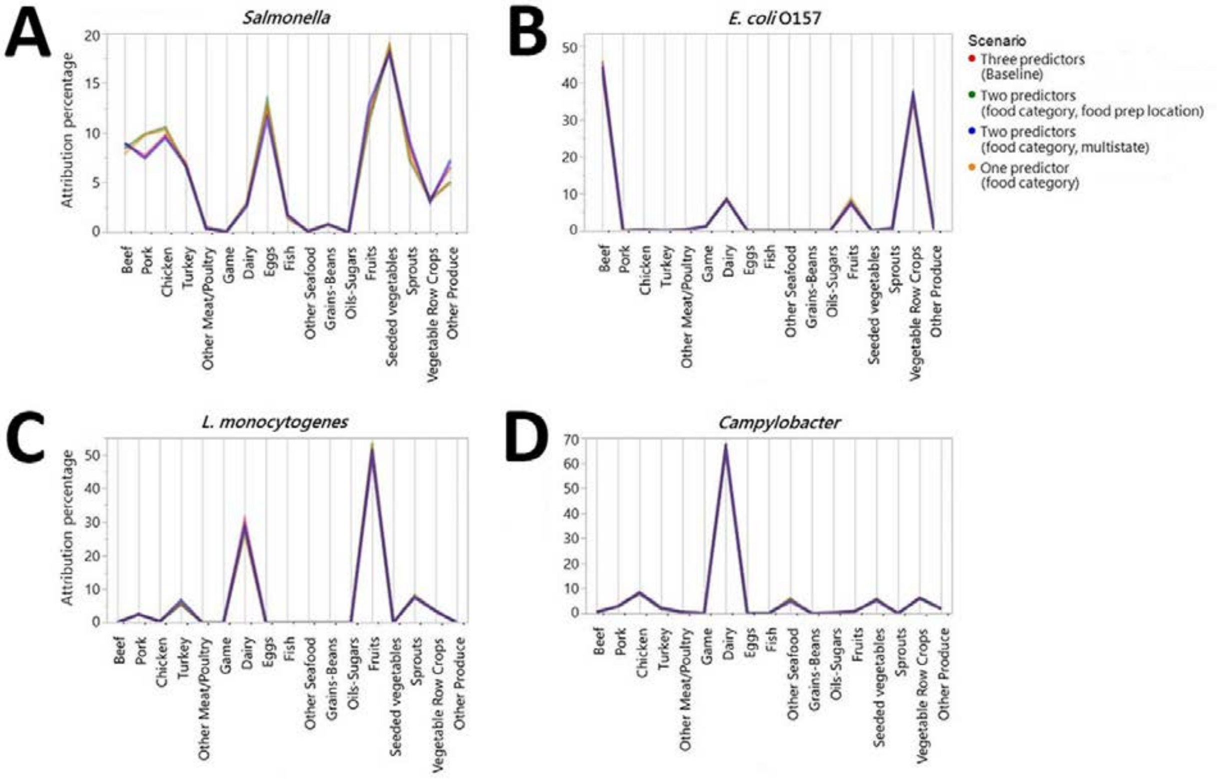

Appendix Figure 8. Estimated percentages of illnesses attributed to food categories under alternative ANOVA modeling scenarios for *Salmonella*, *E. coli* O157, *L. monocytogenes*, and *Campylobacter*, based on analysis of single pathogen, single food category outbreaks – Foodborne Disease Outbreak Surveillance System, 1998–2012. Each panel shows attribution estimates calculated using model-estimated illnesses from 4 different models for each pathogen. The baseline model specification includes food category, the type of location in which food was prepared, and whether an outbreak occurred in a single or multiple states. Credibility intervals are not shown.

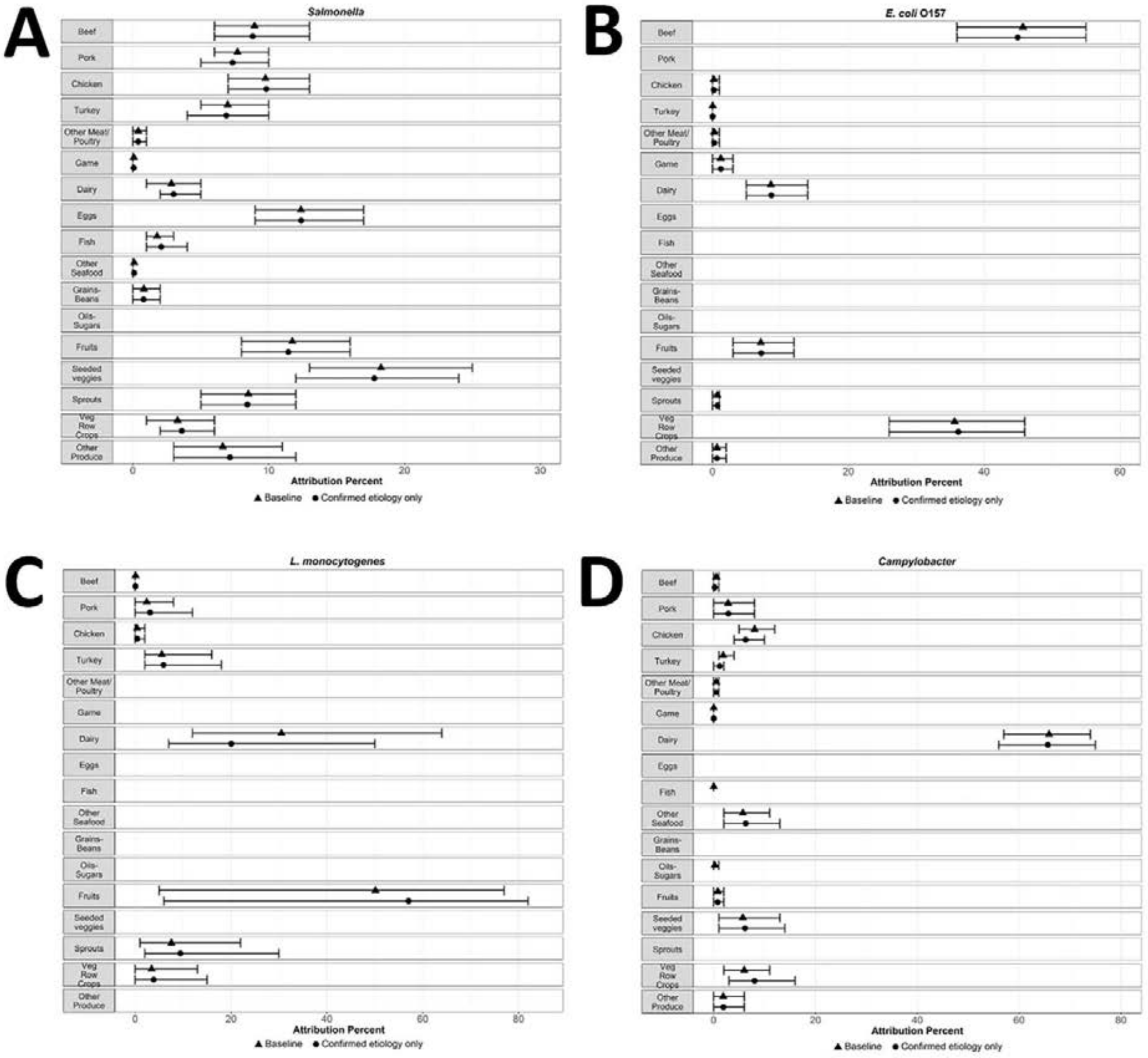

Appendix Figure 9. Comparison of estimated attribution percentages (and 90% credibility intervals) for scenarios including or excluding outbreaks where etiology status is indicated as suspected, for *Salmonella*, *E. coli* O157, *L. monocytogenes*, and *Campylobacter*, based on analysis of single pathogen, single food category outbreaks – Foodborne Disease Outbreak Surveillance System, 1998–2012. Baseline estimates are based on including in the analysis all outbreaks for which etiology status is indicated as being either confirmed or suspected; the alternate scenario is based on including only those outbreaks for which etiology status is indicated as confirmed.

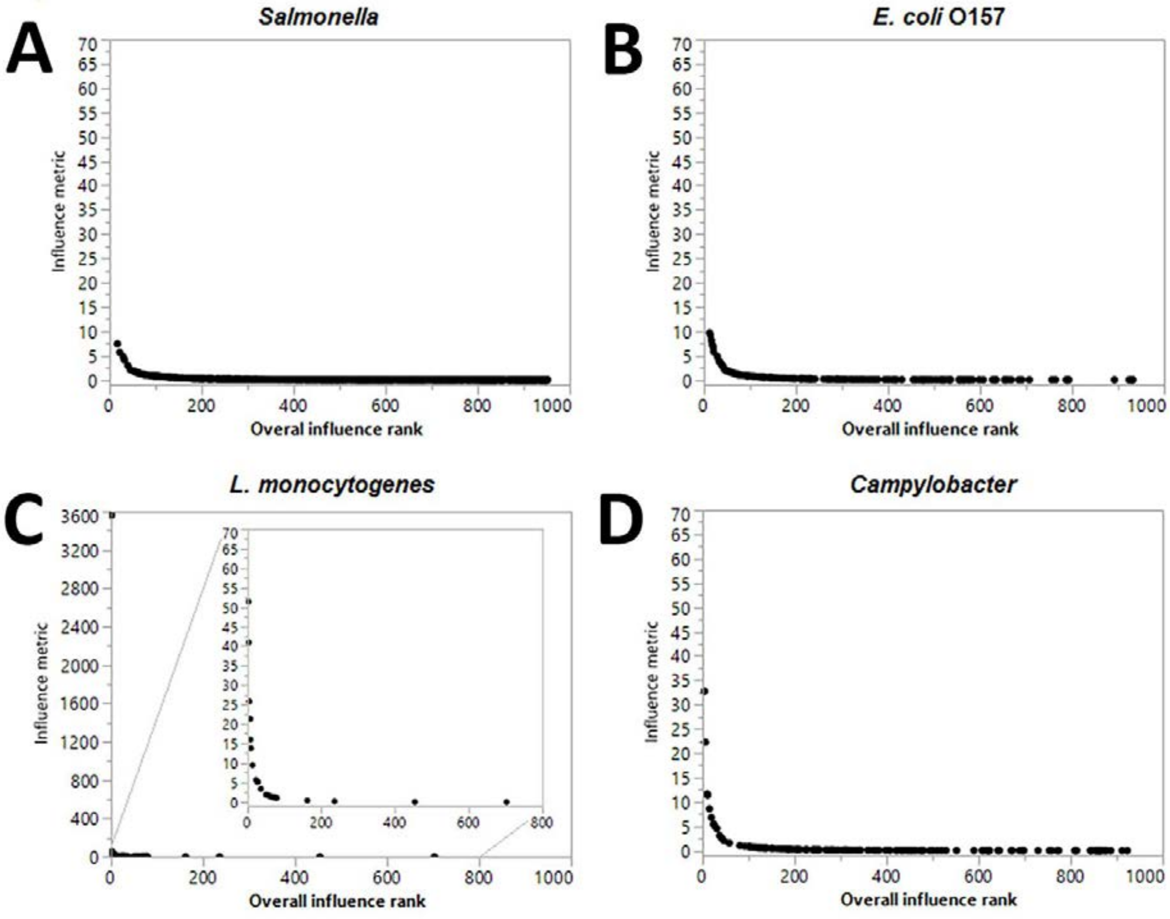

Appendix Figure 10. Calculated influence metric for each outbreak, for *Salmonella*, *E. coli* O157, *L. monocytogenes*, and *Campylobacter*, in descending order, based on analysis of single pathogen, single food category outbreaks – Foodborne Disease Outbreak Surveillance System, 1998–2012. The influence metric is defined as the sum of mean differences squared across all pathogen-food category pairs between the baseline set of attribution estimates and a set of attribution estimates with that outbreak excluded. The overall influence rank of each outbreak is based on the rank order of outbreaks when sorted by the influence metric in descending order. Because *L. monocytogenes* had an extreme value, an inset with the same scale as other pathogens is used to show influence metrics for all other outbreaks.

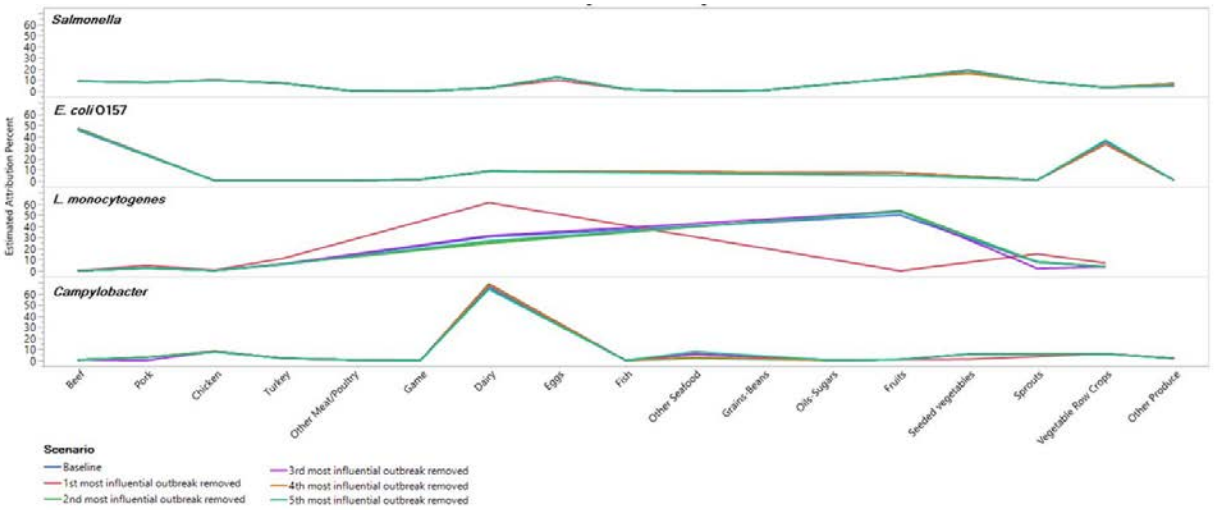

Appendix Figure 11. Impacts of excluding each of the top 5 outbreaks most influential on attribution estimates for *Salmonella*, *E. coli* O157, *L. monocytogenes*, and *Campylobacter*, based on analysis of single pathogen, single food category outbreaks – Foodborne Disease Outbreak Surveillance System, 1998–2012. Each panel shows baseline estimates of attribution percentages compared to estimates for scenarios in which the most influential outbreaks have been excluded. In each scenario, a single outbreak is excluded from the model.
